# Macroporous Directed and Interconnected Carbon Architectures Endow Amorphous Silicon Nanodots as Low-Strain and Fast-Charging Anode for Lithium-Ion Batteries

**DOI:** 10.1007/s40820-023-01308-x

**Published:** 2024-01-29

**Authors:** Zhenwei Li, Meisheng Han, Peilun Yu, Junsheng Lin, Jie Yu

**Affiliations:** 1 Guangdong Provincial Key Laboratory of Semiconductor Optoelectronic Materials and Intelligent Photonic Systems, Shenzhen Engineering Lab for Supercapacitor Materials, School of Material Science and Engineering, Harbin Institute of Technology, Shenzhen, University Town, Shenzhen, 518055 People’s Republic of China; 2 Songshan Lake Materials Laboratory Dongguan, Dongguan, 523808 Guangdong People’s Republic of China; 3 Department of Mechanical and Energy Engineering, Southern University of Science and Technology, Shenzhen, 518055 People’s Republic of China

**Keywords:** Amorphous Si nanodots, Low-strain, Fast-charging, Lithium-ion batteries

## Abstract

Fabricating low-strain and fast-charging silicon-carbon composite anodes is highly desired but remains a huge challenge for lithium-ion batteries. Herein, we report a unique silicon-carbon composite fabricated by uniformly dispersing amorphous Si nanodots (SiNDs) in carbon nanospheres (SiNDs/C) that are welded on the wall of the macroporous carbon framework (MPCF) by vertical graphene (VG), labeled as MPCF@VG@SiNDs/C. The high dispersity and amorphous features of ultrasmall SiNDs (~ 0.7 nm), the flexible and directed electron/Li^+^ transport channels of VG, and the MPCF impart the MPCF@VG@SiNDs/C more lithium storage sites, rapid Li^+^ transport path, and unique low-strain property during Li^+^ storage. Consequently, the MPCF@VG@SiNDs/C exhibits high cycle stability (1301.4 mAh g^−1^ at 1 A g^−1^ after 1000 cycles without apparent decay) and high rate capacity (910.3 mAh g^−1^, 20 A g^−1^) in half cells based on industrial electrode standards. The assembled pouch full cell delivers a high energy density (1694.0 Wh L^−1^; 602.8 Wh kg^−1^) and an excellent fast-charging capability (498.5 Wh kg^−1^, charging for 16.8 min at 3 C). This study opens new possibilities for preparing advanced silicon-carbon composite anodes for practical applications.

**Supplementary Information:**

The online version contains supplementary material available at 10.1007/s40820-023-01308-x.

## Highlights

- MPCF@VG@SiNDs/C, constructed by uniformly dispersing amorphous Si nanodots in carbon nanospheres that are welded on the wall of the macroporous carbon frameworks by vertical graphene, is synthesized and has achieved a few kilogram production per batch.
- Finite element imitation reveals that amorphous Si nanodots with high dispersity in carbon nanosphere can achieve ultra-low stress and strain values during lithiation.
- Unique low-strain property and fast-charging capability are achieved under industrial electrode conditions.

**Supplementary Information:**

The online version contains supplementary material available at 10.1007/s40820-023-01308-x.

## Graphical abstract

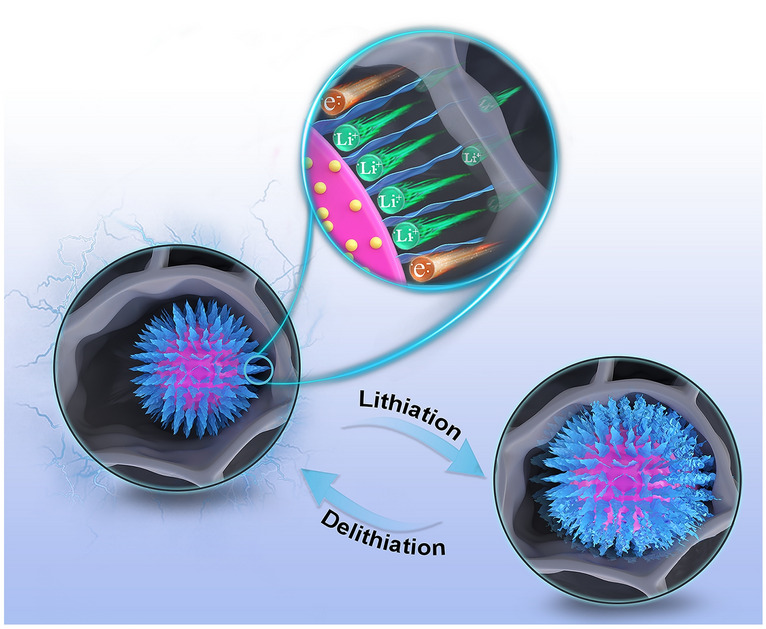

## Introduction

Fast-charging lithium-ion batteries (LIBs), along with high energy density and long cycling life, are highly needed to shorten charging time and extend operating time of electronic mobile devices and electric vehicles [1–3]. However, commercial graphite anodes limit further enhancement of the fast-charging capability of LIBs due to anisotropic lithium storage behavior, which also limit the further improvement of the energy density of LIBs due to low theoretical capacity (372 mAh g^−1^). Silicon (Si) has been considered as the most promising alternative for next-generation LIB anodes due to its isotropic lithium storage path, the highest theoretical specific capacity (~ 4200 mAh g^−1^) among the reported anodes, low operating potential (< 0.5 V vs. Li/Li^+^) [4], and abundant resources [5]. However, Si holds a low intrinsic conductivity (~ 10^−4^ S m^−1^) ascribed to its semiconducting properties, significantly hindering electron transport in electrodes. Moreover, Si suffers from huge volume change (~ 400%) during lithiation/delithiation processes, which unavoidably leads to pulverization of Si particles, unstable solid electrolyte interphase (SEI) layers, and serious interparticle electrical contact failures [6]. These two issues of Si result in the poor fast-charging capability of LIBs along with bad cycling performance. To address these problems, porous Si, nanostructured Si, and Si-based composites have been extensively studied and developed [7–12]. Among them, silicon-carbon composite anodes demonstrate good comprehensive electrochemical performances and have been partially commercialized.

Generally speaking, the C component can support electron transport pathways and buffer the volume change of Si. The core–shell composites with pre-planted void space are widely designed for silicon–carbon anodes [13–17]. As a typical representative of the latest progress, crystalline Si nanodots were uniformly embedded in porous carbon matrix as anode material for LIBs [17]. Ideally, the porous carbon matrix not only can improve the electrical conductivity of the composites but also provide the pre-planted void space to accommodate the volume expansion of Si, thus reducing the swelling of the whole electrode. Although excellent cycle stability can be achieved, the rate capacity is still unsatisfactory. This is mainly due to poor charge transfer at the interfaces between internal Si and external conductive carbon matrix ascribed to the existence of void space. In addition, previous reports have proved that carbon can act as a catalyst to accelerate the reaction between Si and LiPF_6_, which can rapidly consume the electrolyte [18]. That is, an uncompacted carbon shell or a shell damaged during cycling would expose both Si and carbon to the electrolyte, causing accelerated performance decay of the battery. To obtain a compact and robust carbon shell as the electrolyte isolation layer, silicon-carbon composites are commonly modified with multi-level carbon coatings including graphene, amorphous carbon matrix, graphite, etc. [19–21]. However, such designs may lead to poor Li-ion transport capability due to the tortuous and long Li-ion transmission paths in multilevel carbon coatings. Therefore, an ideal carbon structure design needs to simultaneously meet the requirements of accommodating the volume variation of Si to minimize swelling of the whole electrode, isolating Si from the electrolyte during repeated expansion/contraction processes, and ensuring directed and fast Li^+^/electron transport at the Si/C interfaces.

Si offers the main capacity for the silicon-carbon composite anodes by forming Li–Si alloy [22]. Three points about Si need to be noted for the silicon-carbon composite anode design. (1) Particle size. The electrochemical performances of Si are highly dependent on its particle size [23, 24]. Previous reports demonstrated a critical particle size (150 nm) of Si, above which the particle fracture behavior occurs because of the large volume expansion/contraction caused by lithiation/delithiation [23]. In addition, the volume expansion of Si proceeds from the outside to the inside and is accompanied by a continuous accumulation of stress, resulting in the maximum stress inside the Si particles [25]. It is desired that the obtained Si particles should be lower than the critical size, preferably reduce to the nanodot level, to minimize stress accumulation. For instance, Ryu et al. reported the thermal decomposition of tetramethylsilane to make molecular level mixture of Si–C composites, which delivered excellent cycling stability [24]. (2) Si nanoparticle agglomeration. Small particle size (below the critical size) of Si does not necessarily lead to satisfactory electrochemical performance. This is because particle agglomeration occurs in the electrode preparation process due to the large specific surface area of Si nanoparticles. During long-term cycling, with repeated volume expansion/contraction, the agglomeration of Si nanoparticles may reappear [26]. Large-scale agglomeration makes the design of nanosized Si meaningless, which results in serious stress concentration to cause electrode pulverization and failure of electrical contact. (3) Crystallinity of Si. For the commonly used crystalline Si, lithium insertion-induced swelling occurs more often on < 110 > crystal plane, and the rate of swelling on < 110 > plane is even eight times higher than that on < 111 > plane. This tends to cause stress concentration to damage the electrode structure [27]. Fortunately, amorphous Si with an isotropic expansion rate can perfectly avoid this disadvantage of crystalline Si. Considering the design principles of the silicon-carbon composite anodes mentioned above, the construction of rational amorphous Si nanodots/carbon composites is a highly promising solution to minimize stress concentration/electrode swelling during lithiation and ensure directed and fast Li^+^/electron transport at the silicon-carbon interfaces. However, such studies have not been reported so far, and the design of reliable amorphous Si nanodots/carbon composite structures is a daunting challenge and of great significance.

Here, we report a unique silicon-carbon composite fabricated by uniformly dispersing amorphous Si nanodots (SiNDs) in carbon nanospheres (SiNDs/C) that are welded on the wall of the macroporous carbon framework (MPCF) by vertical graphene (VG), labeled as MPCF@VG@SiNDs/C, featuring low-strain property and fast-charging capability for LIB anode. Such a unique structure has a set of attractive advantages: (1) Uniform stress distribution. Amorphous SiNDs with high dispersity in carbon nanosphere, small cumulative stress, and isotropic volume expansion rate during the lithium-insertion process effectively minimize the stress concentration of the MPCF@VG@SiNDs/C upon cycling. (2) Low-strain property. The MPCF allows the volume expansion/contraction of ultra-small SiNDs without obvious volume variation in MPCF@VG@SiNDs/C during cycling. (3) Excellent fast-charging capability. VG connects the inner SiNDs to the outer MPCF, which not only perfectly solves the problem of poor interfacial electron transfer in typical core–shell silicon-carbon composite with pre-planted void space, but also accelerates Li^+^ transport due to the directed and continuous channels. (4) High stability. Macroporous directed and interconnected carbon architecture design suppresses the agglomeration of SiNDs, enhances the structural stability of MPCF@VG@SiNDs/C, and prevents direct exposure of SiNDs to electrolyte upon long-term cycling. (5) High energy density. High electrode compaction density and high capacity allow the prepared cells to have a high energy density. As a result, the MPCF@VG@SiNDs/C electrode exhibits an outstanding fast-charging capability in both half coin cells and pouch full cells, which also possess high energy density and excellent cycle life.

## Experimental Section

### Preparation of SiNDs/C, VG@SiNDs/C, and MPCF@VG@SiNDs/C

#### Preparation of SiNDs/C

First, a controllable heating unit for the evaporation of octamethylcyclotetrasiloxane (C_4_H_16_O_4_Si_4_, OMCTS) was connected to the gas-conducting inlet of the box furnace. The amount of OMCTS vapor in the box furnace was controlled by means of a flow meter and a switching valve. The box furnace was evacuated with Ar for repeated three times and then was heated to 1000 °C. Subsequently, the OMCTS was heated to 145 °C to produce OMCTS vapors, which were introduced into the box furnace (1.2 L min^−1^, flow rate). Meanwhile, H_2_ was also introduced into the box furnace (4.8 L min^−1^, flow rate) and held for 3 h. Last, the box furnace was naturally cooled to room temperature to obtain SiNDs/C.

#### Preparation of VG@SiNDs/C

The prepared SiNDs/C was transferred to a rotary-sealed box furnace. The rotary-sealed box furnace was evacuated three times to remove air and then was heated to 1000 °C in Ar flow. CH_4_ and H_2_ were then introduced into the rotary-sealed box furnace (1.5 L min^−1^, CH_4_; 4.5 L min^−1^, H_2_; flow rate) and were held for 3 h. Last, the furnace was naturally cooled to room temperature to obtain VG@SiNDs/C.

#### Preparation of MPCF@VG@SiNDs/C

Firstly, 2.5 kg of chitosan was dissolved in 100 L of deionized water; then, 5 L of ethanoic acid was added to the above solution, followed by 4 kg of VG@SiNDs/C. The above solution was uniformly stirred and dried at 90 °C for 12 h and then was transferred to a graphite boat and placed in a carbonization furnace, which was heated to 900 °C for 3 h in Ar flow. The MPCF@VG@SiNDs/C was obtained after natural cooling to room temperature. Before it was used as LIB anode, it needed to be crushed to obtain the expected particle size via an airflow crusher.

### Characterizations

Scanning electron microscope (SEM, Philips XL30 FEG), transmission electron microscopy (TEM, FEI F200X, 200 kV), special aberration-corrected transmission electron microscope (AC-TEM, Thermo Fisher Spectra 300, 300 kV), X-ray diffraction (XRD, PANalytical X-ray diffractometer), Raman spectroscopy (Renishaw-200 visual Raman microscope), and X-ray photoelectron spectroscopy (XPS, VG ESCALAB 220i-XL), as well as thermogravimetric analysis (TGA, STA449C) were used to detect the structure and chemical composition of the prepared samples. The Brunauer–Emmett–Teller (BET, Autosorb-iQ) was applied to measure the specific surface area and pore size distribution.

### Electrochemical Performance Measurements

The half-cell electrochemical performances of the prepared samples were measured using 2032 coin-type cells with lithium foil as counter/reference electrode. The electrolyte is 1 M LiPF_6_ in a mixture of ethylene carbonate/diethylene carbonate/dimethyl carbonate at a volume ratio of 1:1:1 with 5 wt% fluoroethylene carbonate, and the separator is Celgard 2500 membrane. To prepare the working electrodes, active materials, carbon black, styrene butadiene rubber, and sodium carboxymethyl cellulose were mixed with a weight ratio of 96:1:1.5:1.5 for MPCF@VG@SiNDs/C and 80:10:5:5 for SiNDs/C and VG@SiNDs/C, which were then cast on the copper foil and dried at 80 °C for 12 h in a vacuum oven. The slurry ratio in the preparation of MPCF@VG@SiNDs/C electrode is different from those of SiNDs/C and VG@SiNDs/C electrodes due to their different specific surface area (detailed explanations are shown in Supporting Information). The average mass loadings of active materials in SiNDs/C, VG@SiNDs/C, and MPCF@VG@SiNDs/C electrode slices were 1.78, 2.11, and 2.52 mg cm^−2^, respectively, which was to ensure the same areal capacity of ~ 3.81 mAh cm^−2^ for testing. The coin cells were assembled in an Ar-filled glovebox. Land CT2001A battery-test system was used to test the electrochemical properties. Electrochemical impedance spectroscopy (EIS; 100 kHz to 0. 01 Hz; 5 mV, amplitude) and cyclic voltammetry (CV; 0.1–20.0 mV s^−1^, scanning rate) tests were carried out on a CHI 760D electrochemical workstation. EIS and CV profiles were obtained at room temperature. Cycling and rate testing were performed in a constant temperature testing cabinet (30 °C). For the NCM811||MPCF@VG@SiNDs/C pouch full cells assembly, the NCM811 and MPCF@VG@SiNDs/C electrodes were used as cathode and anode, respectively. Mixture and coating of 84 wt% NCM811 (HEC Pharm Corporation, China), 8 wt% acetylene black, and 8 wt% polyvinylidene fluoride on aluminum foil were performed to prepare the cathode. The active mass loading of the NCM811 electrode was set to 17.24 mg cm^−2^ to keep the N/P ratio of 1.08. Both electrodes have undergone a calendering process. The prepared cathode and anode electrodes are trimmed to dimensions suitable for pouch cell assembly, with the cathode and anode alternately stacked in a total of 26 layers. The final pre-sealing of the completed pouch cell, following the filling of the electrolyte, was carried out inside a glovebox. Within the pouch cell, the electrolyte was allowed to wet at 40 °C for a duration of 72 h before testing. The capacity of the assembled NCM811|| MPCF@VG@SiNDs/C pouch full cell is 2 Ah. The dimension of the pouch full cells is 55.0 × 40.5 × 3.0 mm. The voltage window is from 2.8 to 4.2 V, and the current densities are from 0.1 to 3 C (1 C = 200 mA g^−1^).

### Simulation Method

In this simulation, the structural mechanics physic field in COMSOL Multiphysics software was used to simulate two different material structures in the microscopic 2D form. The implicit calculation model was that the lithium inlay expansion of random silicon crystal surface particles in carbon sphere particles brought about hard edges of stress, and the overall conservation equation met Newton’s second law:

1 $$0 = \nabla \cdot S + F_{{\text{V}}}$$

where *S* is the stress tensor and *F*_V_ is the volume force.

The method of spatial coordinate randomization was used to randomly generate silicon spheres at any space angle within the radius of carbon spheres on the basis of 3*d* spherical coordinate system. Based on Cartesian coordinate rotation method, Cartesian reference coordinate system with different directions was randomly assigned to each particle to simulate the randomness of anisotropic direction.

For different silicon sphere particles, there are different anisotropic strain expansions:

$$\varepsilon_{111} = \left[ {\begin{array}{*{20}c} 4 & 0 & 0 \\ 0 & 4 & 0 \\ 0 & 0 & 4 \\ \end{array} } \right]$$

$$\varepsilon_{110} = \left[ {\begin{array}{*{20}c} {32} & 0 & 0 \\ 0 & {32} & 0 \\ 0 & 0 & 0 \\ \end{array} } \right]$$

The material properties related parameters are: carbon ball pattern modulus of 60 GPa, Poisson ratio of 0.25, density of 2600 kg m^−3^; the spherical modulus is 1700 GPa, Poisson’s ratio is 0.28, and the density is 2329 kg m^−3^. In the setting of boundary conditions, the outer boundary of the ball is the free surface, and the inner symmetric surface is the roller support setting.

## Results and Discussion

### Materials Synthesis and Characterizations

**Fig. 1:**
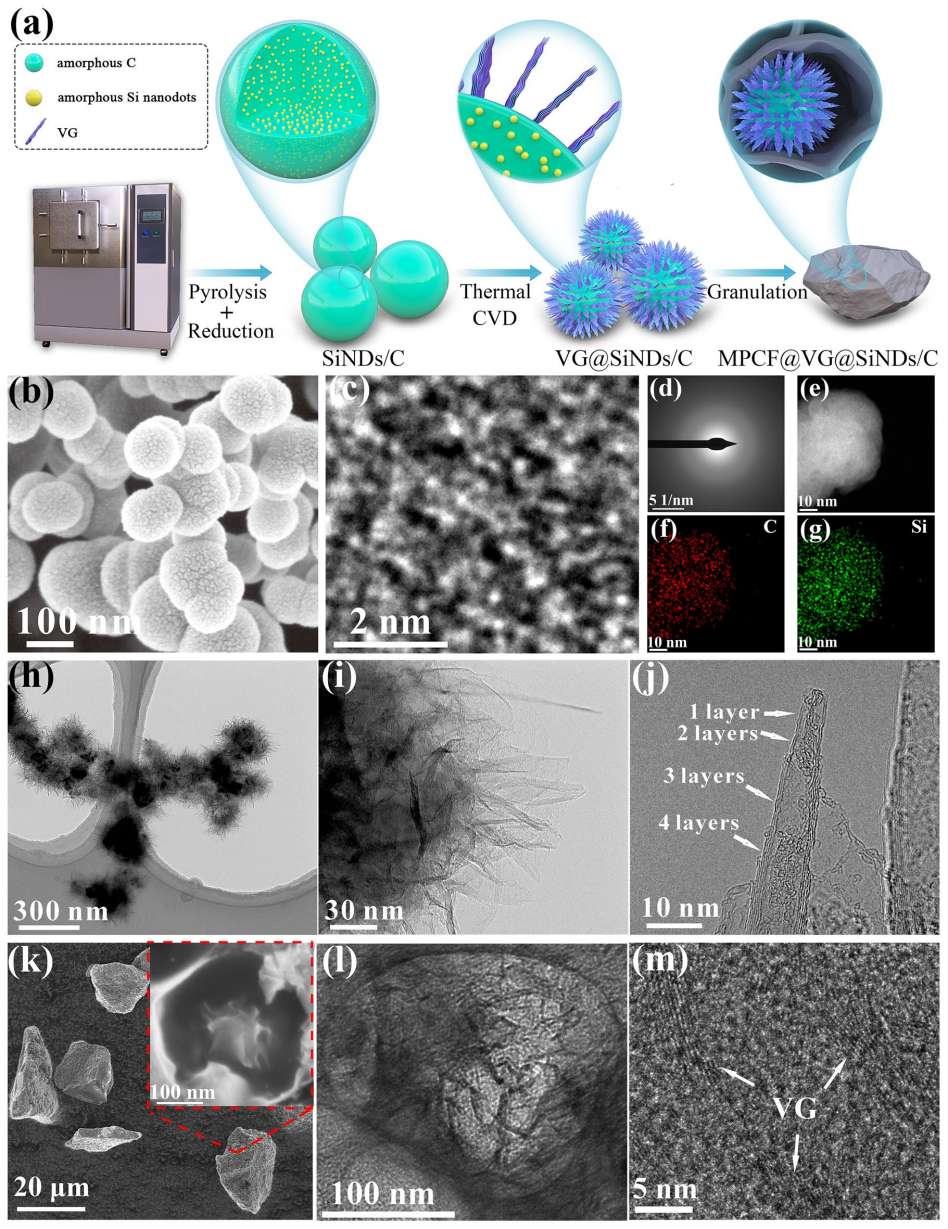
Structural characterizations of samples. **a** Schematic of the synthesis process of samples. **b** SEM, **c** AC-TEM, **d** SAED, **e** HAADF, and **f, g** AC-TEM EDS mapping images of the SiNDs/C. **h, i** TEM and **j** HRTEM images of the VG@SiNDs/C. **k** SEM (inset is a high magnification of **k**), **l** TEM, and **m** HRTEM images of the MPCF@VG@SiNDs/C

Figure 1a displays a schematic of the sample preparation. Firstly, OMCTS was pyrolyzed and reduced to SiNDs/C nanospheres with a high temperature and reducing gases of H_2_ in a static sealed box furnace (Fig. S1). The selection of OMCTS as the source of Si and C is based on a comprehensive assessment of three key factors: (1) OMCTS possesses a reasonable O/Si atomic ratio, and its pyrolysis products have been demonstrated to have high capacity and excellent cycling stability [28], (2) OMCTS is a cost-effective and widely used chemical feedstock, and (3) OMCTS’s relatively low boiling point (175 °C) and mild corrosiveness are conducive to production scaling. Afterward, VG was grown on SiNDs/C nanosphere surface under H_2_ and CH_4_ atmosphere at 1000 °C through the typical thermal chemical vapor deposition (CVD) method in a rotary sealed box furnace (Fig. S2). We define the sample obtained in this process as VG@SiNDs/C. Subsequently, VG@SiNDs/C, chitosan, and ethanoic acid were mixed in deionized water in a certain mass ratio and then were transferred to a sealed carbonization furnace (Fig. S3) to heat to 900 °C under Ar atmosphere to complete the granulation followed by pulverization via an airflow crusher (Fig. S4). Chitosan and ethanoic acid acted as carbon precursors and pore-forming agents, respectively, to construct the MPCF during this process. Meanwhile, VG@SiNDs/C was firmly welded to the wall of the MPCF during the heat treatment process to obtain the MPCF@VG@SiNDs/C of approximately 4.9 kg per batch production (Fig. S5). Note that both the thermal decomposition of OMCTS and the growth of VG are carried out within mass production equipment, while granulation and crushing are typical processes for fabricating commercial Si/C anode materials. Compared to the intricate manufacturing steps involved in the preparation of commercially viable Si/C anodes, the MPCF@VG@SiNDs/C preparation method, while lacking a significant advantage, is relatively straightforward and feasible for large-scale production. Furthermore, all the chemical reagents utilized throughout the entire preparation process, including OMCTS, CH_4_, H_2_, chitosan, and acetic acid, are common and cost-effective materials in industrial production. In light of the above analysis, we believe that the MPCF@VG@SiNDs/C preparation process is a cost-efficient approach suitable for large-scale industrial production, offering potential practical applicability.

SEM and TEM images (Figs. 1b, S6, and S7) show that the SiNDs/C sample consists of uniform nanospheres with an average diameter of ~ 150 nm. Since the contrast increases with the atomic number in TEM observation [28], the dark and bright regions correspond to Si and C, respectively. The average size of SiNDs is about 0.7 nm in the AC-TEM observation (Fig. 1c). To further demonstrate the size of the SiNDs, NaOH is used to etch the SiNDs in the SiNDs/C sample. As shown in Fig. S8, the SiNDs/C retains its spherical structure and shows a microporous structure with a pore size centered at ~ 0.8 nm after etching, denoting that the size of SiNDs is about 0.8 nm, which is basically consistent with the AC-TEM result. The AC-TEM image in Fig. 1c also demonstrates that the silicon and carbon components in the SiNDs/C are amorphous. This result is further confirmed by the selected area electron diffraction (SAED) pattern in Fig. 1d. In addition, high-angle annular dark-field (HAADF) and corresponding AC-TEM EDS elemental mapping images (Fig. 1e–g) suggest that SiNDs are uniformly distributed in the SiNDs/C nanospheres. It is worth noting that the amorphous property, ultrasmall size, and high dispersity of SiNDs enable isotropic volume expansion rate and small cumulative stress upon cycling, thereby ensuring uniform stress distribution and stable structure. Moreover, the amorphous carbon matrix wraps around the amorphous SiNDs, which can act as an electrolyte isolation layer and avoid the agglomeration of SiNDs. After growing VG on SiNDs/C surface, SEM (Fig. S9) and TEM (Fig. 1h) images of VG@SiNDs/C show that VG is uniformly armored on the surface of SiNDs/C. The growth mechanism of VG on SiNDs/C is depicted as follows: initially, carbon atoms generated by the thermal decomposition of CH_4_ deposited on the SiNDs/C surface to form carbon layers. Simultaneously, these carbon layers underwent random etching by H_2_ to result in the formation of defects, steps, and protrusions, which served as nucleation sites for VG [29]. Once graphene flakes were formed, they grew randomly in various directions. When one graphene flake collided with another, a curling force perpendicular to the substrate was generated [29]. This force caused the topmost carbon layers to protrude upward and grew vertically relative to the substrate [30]. With further deposition of carbon atoms, the height and thickness of the carbon layers increased. Concurrently, etching of the carbon layer by H_2_ proceeded. In contrast to the carbon layers at the substrate, the upper carbon layers, due to their more extensive exposure, experienced more vigorous H_2_ etching, resulting in the gradual reduction of graphene layers from the substrate toward the top, ultimately forming a conical structure of VG [29, 31]. In addition, the height of VG is about 50 nm, as confirmed in Fig. 1i. High-resolution (HR) TEM image (Fig. 1j) exhibits that VG with 1–4 atomic layers are perpendicular to the surface of SiNDs/C. The few-layered structure endows good flexibility for VG, which ensures the integrity of the structure even when large strains occur. Figure 1k shows that MPCF@VG@SiNDs/C has a micron-sized and irregular blocky morphology after the granulation and pulverization process. The average size is about 20 μm, in agreement with the results obtained from the laser particle sizer (Table S1). In the high-magnification SEM images (inset of Figs. 1k and S10), the MPCF@VG@SiNDs/C possesses a macroporous honeycomb-like structure with an average pore size of ~ 350 nm. Each honeycomb contains 1–3 VG@SiNDs/C that are welded on the walls of the MPCF by VG (Fig. S10b–d). Such an unique structure is further evidenced by TEM observation (Fig. 1l). The granulation process with different acetic acid/chitosan ratios was also conducted. Sample without added acetic acid displays dispersed VG@SiNDs/C in the carbon matrix, yet no distinct macroporous honeycomb-like structures are observed (Fig. S11a, b), highlighting the role of acetic acid as a porogen in this system. The introduction of a small amount of acetic acid results in the formation of a limited number of macroporous pores (acetic acid/chitosan ratio, 2.5 L/2.5 kg), with the majority of VG@SiNDs/C still disperse in the pore-free carbon matrix (Fig. S11c and d). Increasing the acetic acid/chitosan ratio (5 L/2.5 kg) significantly enhances the number of macroporous pores, and VG@SiNDs/C is uniformly distributed in these pore structures. However, a further increase in the acetic acid/chitosan ratio (7.5 L/2.5 kg) reveals an increase in the number of pores in the sample, resulting in many pores being unoccupied (without VG@SiNDs/C filling, Fig. S11e, f). This adversely affects both the sample’s mechanical strength and the volumetric energy density of the assembled batteries. Therefore, the acetic acid/chitosan ratio is set to 5 L/2.5 kg during the granulation process. Moreover, Fig. 1m confirms that VG is firmly bonded and embedded in the carbon walls without leaving any void at the VG/carbon framework interfaces. Such strong bonding provides fast and continuous paths for charge transport from the outside carbon wall to the inner SiNDs, avoiding the problem of poor interfacial charge transfer in the typical silicon-carbon core–shell composite with pre-planted void space. In addition, VG can also facilitate Li-ion transport due to its directed channel and high electrical conductivity (~ 10^5^ S m^−1^) [29]. More importantly, the MPCF and the flexible VG provide enough space and buffer against the volume expansion of Si. Furthermore, the EDS mapping images demonstrate the good homogeneity of the MPCF@VG@SiNDs/C (Figs. S12 and S13). As a result, the macroporous directed and interconnected carbon architecture with highly dispersed amorphous SiNDs is successfully constructed.

**Fig. 2:**
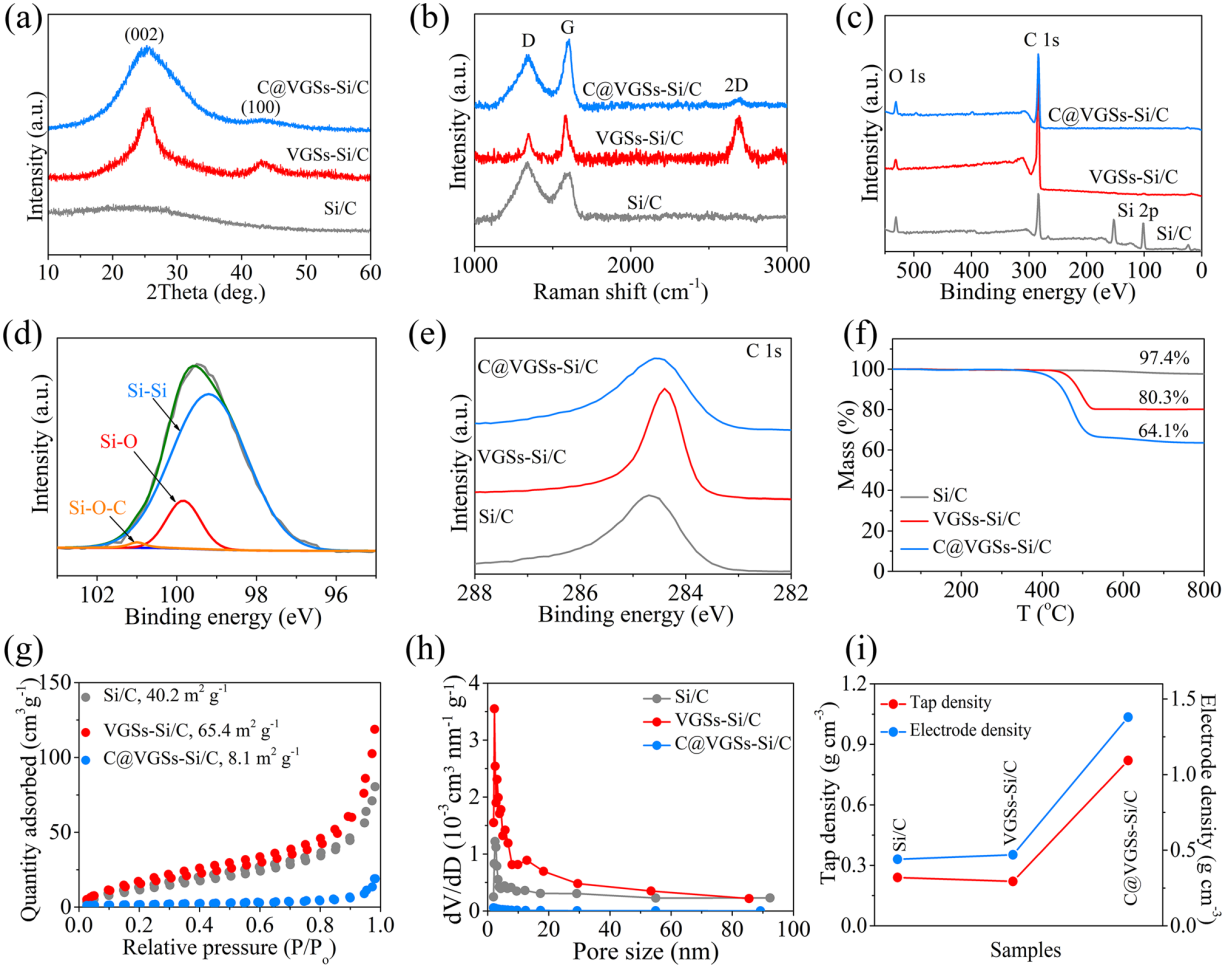
Sample analysis.** a** XRD patterns, **b** Raman spectra, and **c** XPS spectra of SiNDs/C, VG@SiNDs/C, and MPCF@VG@SiNDs/C. **d** Si 2*p* spectrum of SiNDs/C. **e** C 1*s* spectra, **f** TGA spectra, **g** nitrogen adsorption/desorption isotherms, **h** pore size distribution, and **i** tap/electrode density of SiNDs/C, VG@SiNDs/C, and MPCF@VG@SiNDs/C

The crystalline structure of the samples was analyzed by XRD, as exhibited in Fig. 2a. No peaks can be detected in the XRD pattern of SiNDs/C, indicating its amorphous nature, in good agreement with the HRTEM results in Fig. 1c, d. After growing VG on the SiNDs/C, two obvious peaks are located at ~ 25.5° and 43.5°, corresponding to (002) and (100) planes of VG, respectively [29]. In the diffraction pattern of MPCF@VG@SiNDs/C, the reflections for the VG can also be detected, but are relatively broadening. This may be because the amorphous MPCF covers the signals of VG. Figure 2b exhibits Raman spectra of the samples. Two typical carbon peaks are detected in all samples; the G band (~ 1595 cm^−1^) corresponds to the in-plane stretching vibration of *sp*^2^ carbon atoms and the D band (~ 1355 cm^−1^) is related to lattice defects of carbon atoms [31]. The intensity ratio between the D and G bands reflects the defect concentration in the samples [31]. SiNDs/C exhibits a high *I*_D_/*I*_G_ value, indicating the presence of a substantial number of defects, attributed to the presence of amorphous carbon (evidenced by the AC-TEM and XRD results). Upon the growth of VG on the SiNDs/C surface, the low-defect VG significantly reduces the *I*_D_/*I*_G_ value of VG@SiNDs/C. Subsequent granulation of VG@SiNDs/C results in the obscuration of VG signals by the defect-rich amorphous carbon derived from chitosan. This leads to a notable increase in the *I*_D_/*I*_G_ value for MPCF@VG@SiNDs/C compared to VG@SiNDs/C. Apart from the D and G bands, a strong 2D band (~ 2710 cm^−1^) with a low value of *I*_G_/*I*_2D_ (0.98) can be detected for VG@SiNDs/C, demonstrating the few-layered structure of VG [29]. This result is in agreement with the HRTEM results in Fig. 1j.

We performed XPS to study the features of elements and chemical states of the prepared samples. In the full survey XPS spectra (Fig. 2c), SiNDs/C shows the Si 2*p*, C 1*s*, and O 1*s* peaks, evidencing the presence of the Si and C components. The Si content on the sample surface is an important indicator. This is because when a large amount of Si is exposed on the surface of the active materials, it will repeatedly consume the electrolyte [32]. The detection depth of XPS is usually several nanometers, making it an efficient tool for analyzing the elemental content on the sample surface. As can be seen, SiNDs/C has an obvious Si 2*p* peak with a high surface Si content, suggesting that a large number of Si nanodots are exposed on the surface. After being covered by VG and MPCF, the disappeared Si 2*p* peaks of VG@SiNDs/C and MPCF@VG@SiNDs/C demonstrate that there is no exposure of SiNDs on the sample surface, avoiding direct contact between Si and electrolyte. In the high-resolution XPS spectrum of Si 2*p*, the SiNDs/C exhibits a prominent Si–Si peak, a minor Si–O peak, and a weak Si–O–C peak (Fig. 2d), indicating that the primary component of SiNDs/C is Si^0^. The presence of Si–O and Si–O–C is primarily attributed to the incomplete reduction of SiNDs/C. Peak deconvolution of the C 1*s* spectrum was performed to assess the potential existence of Si–C bonds in SiNDs/C. It is evident that, apart from the predominant C–C bonds and a small amount of C–O and C=O bonds, there is no Si–C bond in the C 1*s* spectrum (Fig. S14), suggesting the absence of SiC phase in SiNDs/C. It is worth mentioning that the concentration of H_2_ and the temperature play a pivotal role in determining the composition of pyrolysis products. Pyrolyzing OMCTS in Ar at 1000 °C without H_2_ for reduction results in a significant presence of Si–O in the pyrolysis products (Fig. S15). The introduction of an excess of H_2_ (flow ratio, OMCTS/H_2_, 1/8) leads to a significant reduction in oxygen-containing groups in the pyrolysis products but simultaneously results in the generation of a substantial number of Si–C bonds (Fig. S16a) and SiC phase (Fig. S16b). Pyrolysis conducted at 1000 °C with a moderate H_2_ concentration (flow ratio, OMCTS/H_2_, 1/4) yields SiNDs/C characterized by the absence of Si–C bonds and only a limited presence of oxygen-containing groups. Furthermore, by maintaining a moderate H_2_ concentration (flow ratio, OMCTS/H_2_, 1/4), pyrolysis conducted at 800 °C reveals a significant presence of Si–O and Si–O–C bonds in the pyrolysis products, with the absence of Si–Si bonds (Fig. S17). This is likely due to the lower temperature, which inhibits the reactivity of H_2_. These findings emphasize the importance of moderate H_2_ concentration and a reasonable reaction temperature for obtaining the desired SiNDs/C samples. In C 1*s* spectra of samples (Fig. 2e), VG (C=C, *sp*^2^) on VG@SiNDs/C surface induces a shift toward low binding energy of the C 1*s* peak compared to the amorphous carbon (C–C, *sp*^3^) of the SiNDs/C and MPCF@VG@SiNDs/C samples [33]. Figure 2f displays the TGA profiles of samples. According to the residual weight after heating to 1000 °C in air (Si → SiO_2_; C → CO_2_), the Si contents in SiNDs/C, VG@SiNDs/C, and MPCF@VG@SiNDs/C can be calculated to be ~ 45.5, 37.5, and 29.9 wt%, respectively. Figure 2g, h presents N_2_ adsorption–desorption curves and pore size distribution, respectively, of the prepared samples. All samples display a mesoporous structure, in which the specific surface area of VG@SiNDs/C (65.4 m^2^ g^−1^) is larger than that of SiNDs/C (40.2 m^2^ g^−1^) because of the presence of few-layered VG. After welding VG@SiNDs/C to the walls of MPCF, micron-sized MPCF@VG@SiNDs/C shows a significantly decreased specific surface area of 8.1 m^2^ g^−1^. Therefore, the tap density and electrode compaction density can be as high as 0.82 and 1.38 g cm^−3^, respectively (Fig. 2i and Table S2).

### Electrochemical Performance Characterizations for LIBs

**Fig. 3:**
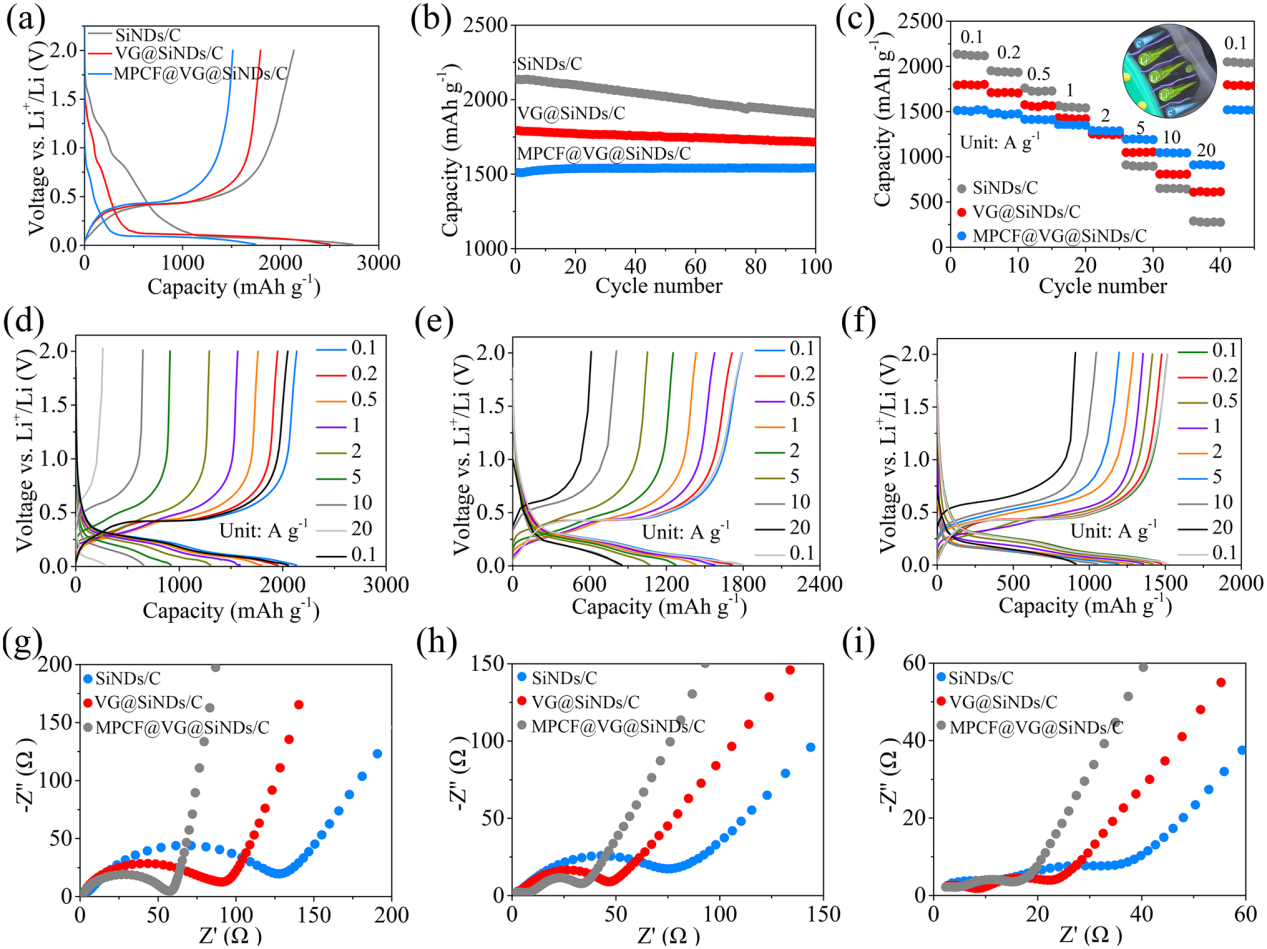
Lithium-ion storage performances. **a** First charge/discharge curves, **b** cycling performance at 0.1 A g^−1^, **c** rate performance, **d–f** charge/discharge curves at different current densities, Nyquist plots **g** before cycling, **h** after the 1st cycle, and **i** after rate test of SiNDs/C, VG@SiNDs/C, and MPCF@VG@SiNDs/C

Figure 3 shows the electrochemical performances of the SiNDs/C, VG@SiNDs/C, and MPCF@VG@SiNDs/C electrodes in coin-type half cells. Note that these electrodes were tested at the same areal capacity of about 3.8 mAh cm^−2^. Different from the SiNDs/C and VG@SiNDs/C electrodes, the MPCF@VG@SiNDs/C electrode can be fabricated under industrial electrode conditions (areal capacity loading ≥ 3.3 mAh cm^−2^, binder ≤ 3 wt%, conductive agents ≤ 1 wt%) [34]. Specific explanations are given in the experimental section in Supporting Information. At a current density of 0.1 A g^−1^, the first charging capacity of SiNDs/C, VG@SiNDs/C, and MPCF@VG@SiNDs/C is 2134.6, 1794.6, and 1512.2 mAh g^−1^, respectively (Fig. 3a). The capacity mainly depends on the amount of Si in the sample (calculated in Fig. 2f). The voltage profile of SiNDs/C is quite different from that of pure Si electrode [9]. It may be attributed to the mixed products like Si, Si–O, Si–O–C, and the nanoscale carbon matrix in SiNDs/C. MPCF@VG@SiNDs/C with the lowest specific surface area delivers the largest initial Coulombic efficiency (CE) of 86.6%. Although the first charging capacity of MPCF@VG@SiNDs/C is slightly lower than SiNDs/C and VG@SiNDs/C, it exhibits the highest capacity retention of 102.0% after 100 cycles, in comparison with 95.7% for VG@SiNDs/C and 89.4% for SiNDs/C (Fig. 3b, Table S3). The lowest capacity retention of SiNDs/C is largely owing to the lack of space for Si expansion and its high Si contents on the particle surface (repeatedly consuming the electrolyte). This can also be confirmed by the significantly increased electrode thickness (57.0%) and the numerous cracks on the electrode surface after cycling (Fig. S18). Growing flexible VG on the SiNDs/C surface not only covers the exposed Si on the particle surface, but also buffers the volume change of the SiNDs/C, thus improving the cycle stability of VG@SiNDs/C. By comparison with SiNDs/C, VG@SiNDs/C also shows a reduced thickness change of 28.4% with little cracks on the surface after cycling (Fig. S19), demonstrating enhanced structural stability. After welding VG@SiNDs/C to the walls of MPCF, MPCF@VG@SiNDs/C with enough expansion space and interconnected carbon structure brings the best cycling stability over other samples. In addition, the MPCF@VG@SiNDs/C electrode remains intact with an ultralow thickness expansion of 1.1% (Fig. S20), suggesting its unique low-strain properties to ensure excellent cycling stability. Figure 3c shows the rate performance of samples assessed by ramping current densities from 0.1 to 20 A g^−1^. At 2 A g^−1^, the capacity of MPCF@VG@SiNDs/C maintains at 1288.6 mAh g^−1^, corresponding to 85.2% of the capacity at 0.1 A g^−1^ (1512.8 mAh g^−1^). Even after cycling at 10 and 20 A g^−1^, MPCF@VG@SiNDs/C still delivers advanced invertible capacities of 1045.4 and 910.3 mAh g^−1^, respectively. Moreover, absolute recovery of the capacity is achieved when the current density returns to 0.1 A g^−1^. By comparison, although delivering high capacity at lower current densities (< 2 A g^−1^), the VG@SiNDs/C and SiNDs/C exhibit far inferior rate capacity than MPCF@VG@SiNDs/C at high current densities (> 5 A g^−1^). Such superior rate capacity of MPCF@VG@SiNDs/C is mainly ascribed to that the few-layered VG: (1) provides directed and continuous channels to accelerate Li^+^ transport and (2) ensures highly efficient charge transfer at the interfaces between internal SiNDs and external MPCF (inset of Fig. 3c). In addition, all these samples remain stable during the rate testing process, as confirmed by the similar charge/discharge profiles at each current density (Fig. 3d–f). To further reveal the reason for the excellent rate capability of MPCF@VG@SiNDs/C, the EIS study was carried out (Fig. 3g–i) and was fitted by the equivalent circuit (Fig. S21). For the Nyquist plots, the diameter of the semicircle reflects the charge transfer resistance (*R*_ct_), the slope of the sloping line corresponds to the Warburg impedance (W) of Li^+^ diffusion, and the value of the intersection with the *x*-axis is related to the electrolyte resistance (*R*_s_) [35]. The corresponding values are listed in Table S4. MPCF@VG@SiNDs/C delivers the lowest *R*_ct_, *R*_s_, and SEI layer resistance (*R*_f_), as well as the largest straight-line slope compared to VG@SiNDs/C and SiNDs/C, indicating superior charge transport capability. It is important to note that the nano-sized VG@SiNDs/C, as opposed to micro-sized MPCF@VG@SiNDs/C, results in a reduced tap density and electrode compaction density (Fig. 2i), thereby causing a notably increased electrode thickness under the same areal capacity conditions (Figs. S19c and S20c). The increased electrode thickness undoubtedly extends the charge transport distance. Additionally, the contact resistance between VG@SiNDs/C particles is heightened due to the nano-sized dimensions of VG@SiNDs/C when compared to micro-sized MPCF@VG@SiNDs/C. Therefore, despite VG@SiNDs/C's larger specific surface area, which results in better wetting between VG@SiNDs/C and electrolyte than that between MPCF@VG@SiNDs/C and electrolyte, the VG@SiNDs/C electrode continues to exhibit a larger interfacial *R*_ct_. These results strongly support the best rate capability of MPCF@VG@SiNDs/C in Fig. 3c. In addition, the *R*_ct_ of these samples continues to decrease during cycling. This is mainly caused by the activation and stabilization of the electrode [36].

**Fig. 4:**
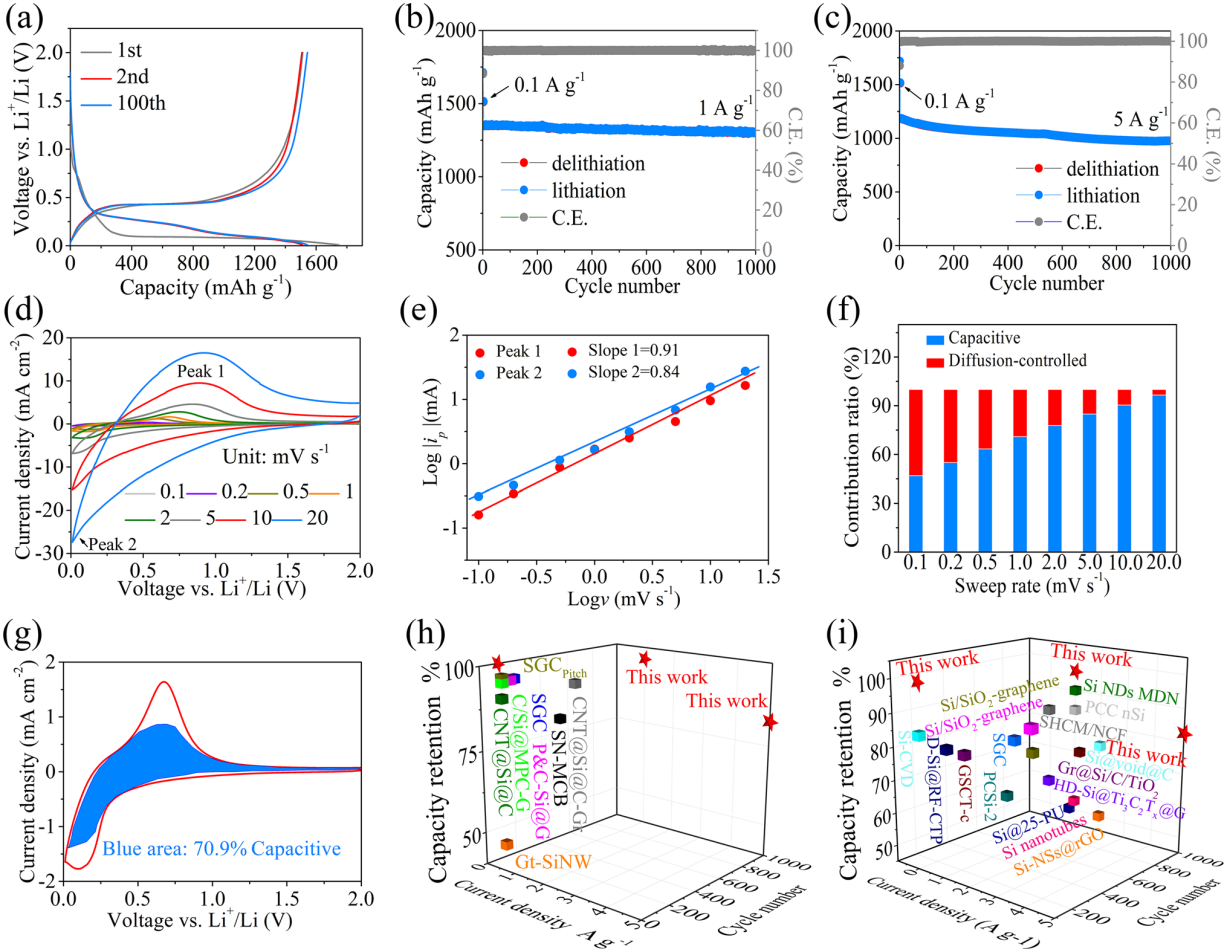
Long-term cycle stability and kinetic analysis of MPCF@VG@SiNDs/C and performance comparison.** a** Charge–discharge profiles at the 1st, 2nd, and 100th cycles at 0.1 A g^−1^, long-term cycling performances at **b** 1 A g^−1^ and **c** 5 A g^−1^, **d** CV profiles at different scan rates, **e** Log *i*_*p*_ against Log *v* at marked peaks, **f** the percentages of pseudocapacitive contribution at different sweep rates, **g** the detailed pseudocapacitive contribution at 1 mV s^−1^. Performance comparison of the MPCF@VG@SiNDs/C with recently reported silicon-carbon anodes based on industrial electrode standards **h** and non-industrial electrode standards **i** for LIBs

At 0.1 A g^−1^, the discharge/charge curves of the 2nd and the 100th cycle overlap almost exactly except for the first cycle (Fig. 4a), suggesting that the electrode is highly reversible during cycles. The difference in the discharge curve in the first cycle with other cycles is mainly ascribed to the formation of the SEI layer [36]. The long-term cycle stability of the MPCF@VG@SiNDs/C electrode is also tested. At 1 A g^−1^ (Fig. 4b), the MPCF@VG@SiNDs/C delivers a high reversible capacity of 1301.4 mAh g^−1^ after 1000 cycles without apparent decay, denoting its excellent cycling performance. Even at a higher rate of 5 A g^−1^ (Fig. 4c), the MPCF@VG@SiNDs/C exhibits a considerable reversible capacity and excellent cycle stability, achieving 975.6 mAh g^−1^ over 1000 cycles with a capacity retention of 82.5%. To comprehend the mechanism of Li^+^ storage kinetics in the prepared electrodes, CV measurements with sweeping rates ranging from 0.1 to 20 mV s^−1^ were taken (Figs. 4d, S22a, and S23a). The capacitive effect of the battery can be calculated based on Eq. (2) [37–40]:

2 $$i = av^{b}$$

where *i* and *v* represent current density and scan rate, respectively. And *a* and *b* are empirical constants, in which the *b* value of 1 represents that the system is controlled by the capacitance. For MPCF@VG@SiNDs/C, *b* values are calculated to be 0.91 and 0.84 for peaks 1 and 2, respectively, demonstrating that the capacitive process dominates Li-ion storage dynamics. In addition, the *b* values of MPCF@VG@SiNDs/C are larger than those of SiNDs/C (0.68, peak 1; 0.62, peak 2; Fig. S22b) and VG@SiNDs/C (0.78, peak 1; 0.74, peak 2; Fig. S23b), suggesting that the MPCF@VG@SiNDs/C electrode possesses higher capacitive contribution on capacity. This may be due to the presence of more interfaces in the three phases (SiNDs/C nanospheres, VG, and MPCF), which can be used for additional lithium-ion storage. The capacitive contribution can be further quantified by Eq. (3) [41–43]:

3 $$i\left( V \right) = k_{1} v + k_{2} v^{1/2}$$

where *i(V)*, *k*_*1*_*v*, and *k*_*2*_*v*^*1/2*^ reflect total current, capacitance process, and diffusion-controlled behavior, respectively. As the scan rate increases from 0.1 to 20 mV s^−1^, the contribution of capacitance rises from 46.9 to 96.5% for the MPCF@VG@SiNDs/C electrode (Fig. 4f), higher than SiNDs/C (30.5–75.4%, Fig. S22c) and VG@SiNDs/C (41.3–87.6%, Fig. S23c). The higher capacitive contribution is beneficial for obtaining higher capacity and better rate capability. The detailed pseudocapacitive contribution at 1 mV s^−1^ for MPCF@VG@SiNDs/C is presented in Fig. 4g. These results demonstrate that the synergistic effect of macroporous directed and interconnected carbon architectures and amorphous SiNDs significantly boosts the Li-ion transport and storage in the MPCF@VG@SiNDs/C electrode. In addition, the electrochemical performances of the MPCF@VG@SiNDs/C electrode and previously reported Si-based anodes under industrial electrode standards are shown in Fig. 4h and Table S5, which can conclude that the MPCF@VG@SiNDs/C displays the best cycle stability and rate performance. Even when compared to non-industrial electrodes with excessive amounts of conductive agents and binders, as well as low areal capacity, our MPCF@VG@SiNDs/C still shows significant advantages in terms of cycle stability and rate performance (Fig. 4i and Table S6).

**Fig. 5:**
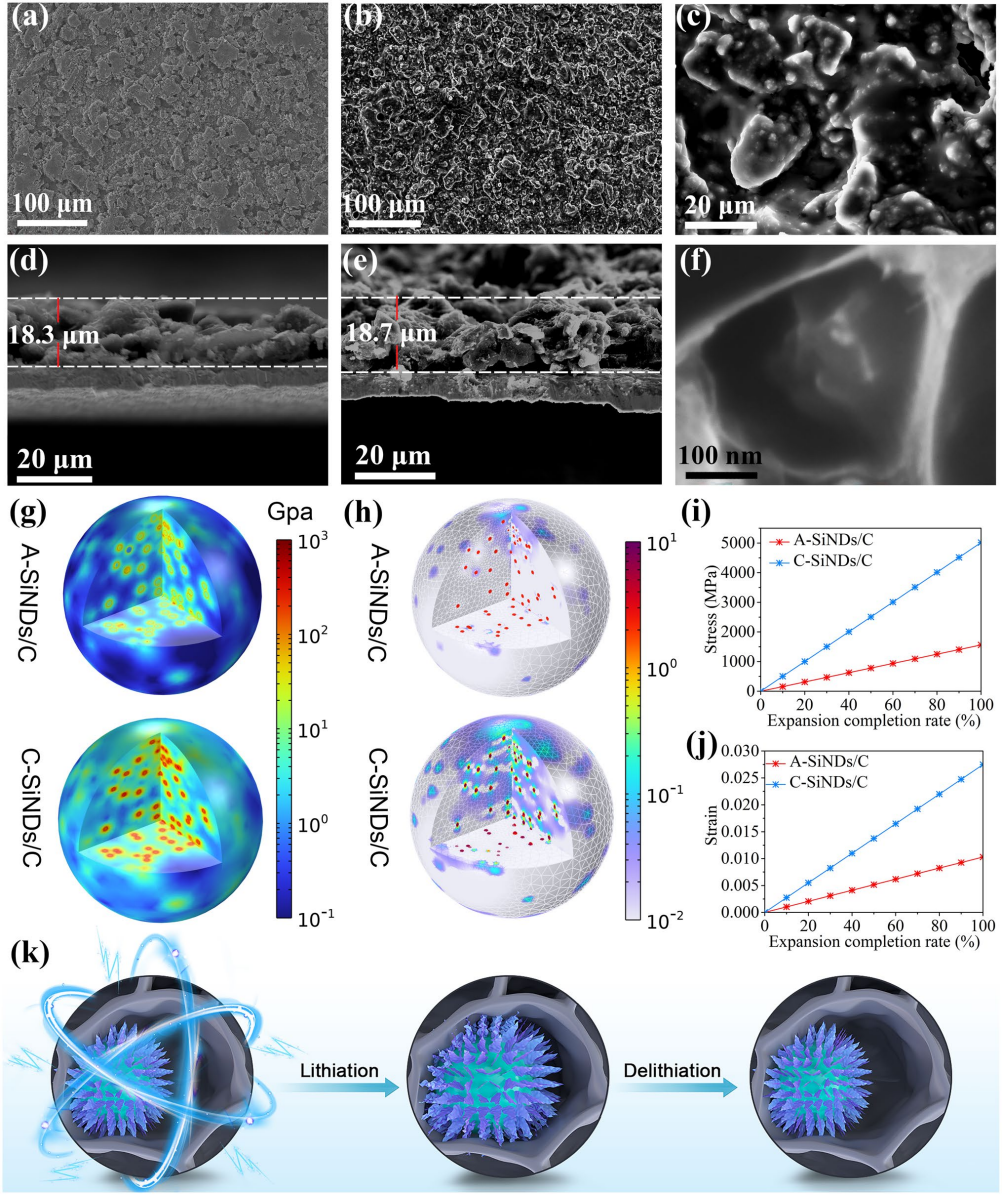
Low-strain property characterization. Top-view SEM images of the MPCF@VG@SiNDs/C electrodes **a** before cycling and **b, c** after 1000 cycles at 1 A g^−1^. Side-view SEM images of the MPCF@VG@SiNDs/C electrodes **d** before cycling and **e** after 1000 cycles at 1 A g^−1^. **f** High-magnification SEM images of the MPCF@VG@SiNDs/C after 1000 cycles at 1 A g^−1^. **g** Stress distribution over A-SiNDs/C and C-SiNDs/C after volume expansion up to 400%. **h** Volumetric strain of A-SiNDs/C and C-SiNDs/C after volume expansion up to 400%. **i** Average stress and **j** strain variation of A-SiNDs/C and C-SiNDs/C during the expansion process. **k** Schematic diagram of the low-strain property

To further comprehend the excellent cycling stability of the MPCF@VG@SiNDs/C electrode, we further observed its morphology and electrode swelling. Figure 5a shows that the MPCF@VG@SiNDs/C electrode has a compact surface morphology before cycling. After 1000 cycles at 1 A g^−1^, the surface of the electrode remains intact and free of cracks, although it becomes relatively rough (Fig. 5b, c), demonstrating that the long-term and repeated lithiation/delithiation processes do not cause significant damage to the electrode structure. From the side-view SEM images it can be seen that the initial thickness of the MPCF@VG@SiNDs/C electrode is about 18.3 µm before cycling (Fig. 5d). After 1000 cycles, the thickness increases slightly to ~ 18.7 µm (Fig. 5e), showing a small ratio of change of ~ 2.2%. It is suggested that hundreds of volume expansions/contractions of SiNDs in the MPCF do not cause damage of the MPCF@VG@SiNDs/C and the whole electrode structure. This can be further confirmed by the observation in Fig. 5f, as can be seen, the MPCF@VG@SiNDs/C maintains its morphological and structural integrity after 1000 cycles. The thickened VG may be due to the deposition of the decomposition products of the electrolyte or residual binder. These results above denote the unique low-strain property of the MPCF@VG@SiNDs/C, which strongly supports excellent cycling stability and provides a solid foundation for fast-charging capability.

In order to gain further insight into the properties of low strain and uniform stress distribution that ensure high cycling stability of MPCF@VG@SiNDs/C electrodes from a mechanical point of view, we employed a finite element model to evaluate the lithiation-induced stress and strain state. For the mechanical simulations, we chose crystalline and amorphous Si nanodots for comparison. Two idealized models were built for both samples (Fig. S24), i.e., one was that crystalline SiNDs were dispersed in amorphous carbon nanospheres (named C-SiNDs/C) and the other was that amorphous SiNDs were dispersed in amorphous carbon nanospheres (named A-SiNDs/C). The Si content in both models was set as 45.5 wt%, which is consistent with the Si content in the SiNDs/C nanospheres in the experimental sample (Fig. 2f). According to previous reports, lithium ions diffused faster along the < 110 > axis than along the < 111 > axis of crystalline Si during lithiation, which resulted in a preferential and pronounced volume expansion in the direction of the < 110 > axis (the expansion rate on the < 110 > crystal plane was eight times higher than that on the < 111 > crystal plane) [27, 44]. Therefore, an anisotropic volume expansion was introduced to the C-SiNDs/C model. Meanwhile, the isotropic expansion rate of the amorphous Si was also set for the A-SiNDs/C model. Figure 5g, h shows the distribution of the effective stress (von Mises stress) and volumetric strain for A-SiNDs/C and C-SiNDs/C models, respectively, in the case of 400% volume expansion of SiNDs. By comparison with the C-SiNDs/C model, the A-SiNDs/C model has a more uniform stress/strain distribution. Moreover, the values of stress and strain of the A-SiNDs/C model are also much lower than those of the C-SiNDs/C model. That is, the stress and strain generation in A-SiNDs/C model is significantly suppressed compared to those in C-SiNDs/C model. Figure 5i, j displays the average stress and volumetric strain changes of the A-SiNDs/C and C-SiNDs/C models during the expansion process, respectively. It can be seen that the average stresses and strain in both models continue to increase as expansion proceeds. Both average stain and stress values of the A-SiNDs/C model are much lower than those of the C–Si/C model throughout the expansion process, signaling that the isotropic volume expansion of amorphous Si greatly relieves the stress and reduces the volumetric strain in the A-SiNDs/C model, which thus withstands the increasing stress and strain during the lithiation process. Accordingly, the simulation results clearly show that our SiNDs/C sample with amorphous SiNDs has higher structural stability than commonly used crystalline Si materials, which provides an effective guarantee for the long-term cycling life of the MPCF@VG@SiNDs/C electrodes. More importantly, the simulation results pave the way for the subsequent development of amorphous SiNDs in silicon-carbon anodes.

To explore the lithium-ion storage mechanism of Si in the MPCF@VG@SiNDs/C, ex situ XRD and XPS were performed at different charge–discharge states (Fig. S25). It should be pointed out that the signal of Si cannot be detected by XPS due to the growth of VG and the existence of the macroporous carbon framework, as displayed in Fig. 2c. Consequently, the SiNDs/C was chosen to explore the lithium-ion storage mechanism of Si in the composite. As depicted in Fig. S25a, the SiNDs/C still maintains its amorphous feature during lithiation and delithiation processes. After discharging to 0.01 V, Si–Si bonds (99.0 eV) in the SiNDs/C completely disappear, being replaced by Si–Li bonds (~ 97.0 eV). Subsequently, as the SiNDs/C electrode is charged to 3 V, Si–Li bonds vanish, accompanied by the re-emergence of Si–Si bonds (Fig. S25b). This indicates that the lithium storage mechanism of amorphous SiNDs in our composite is also a typical alloying process of Si and Li. To analyze the mechanism of stable lithium storage for the MPCF@VG@SiNDs/C electrode, an analysis of the evolution of the SEI film of MPCF@VG@SiNDs/C during the cycling was performed (Fig. S26). Before cycling, the F 1*s* and Li 1*s* spectra display peaks of LiPF_6_ in the electrolyte. After the initial cycle, the appearance of Li_2_CO_3_, R–CH_2_–OCO_2_Li, and C–H in the C 1*s* spectra, along with Li–F bonds in the F 1*s* and Li 1*s* spectra, indicates the formation of the SEI film. Notably, after 100 cycles, the peak shapes in the C 1*s*, F 1*s*, and Li 1*s* spectra nearly overlap with the first cycle. This demonstrates that the composition and content of the SEI film remain largely unchanged during prolonged cycling, confirming the structure stability of the SEI film of the MPCF@VG@SiNDs/C electrode. It is important to note that a stable SEI film is crucial for achieving excellent electrochemical performance [45].

To conclude, the structural design of MPCF@VG@SiNDs/C possesses the following advantages. First, the VG and C components of the SiNDs/C nanosphere as the electrolyte isolation layer can prevent direct exposure of Si nanodots to the electrolyte (confirmed in Fig. 2c). Second, the MPCF allows the volume expansion/contraction of SiNDs/C nanospheres without obvious volume change in MPCF@VG@SiNDs/C, thus enabling unique low-strain property and stable Li-ion storage. Third, VG as a bridge connects the internal SiNDs/C nanosphere and the external MPCF, avoiding the problem of poor interfacial charge transfer in the typical silicon-carbon core–shell composite with pre-planted void space. The good flexibility of VG ensures the integrity of the electron transport channel even after large strains caused by repeated volume expansion/contraction. Last, VG with directed and continuous channels can accelerate Li^+^ transport, which can break the restriction by the tortuous Li-ion diffusion path in the carbon frameworks. To further evidence the contribution of VG on the Li-ion transport in MPCF@VG@SiNDs/C, we directly composited the SiNDs/C nanospheres and the MPCF (named MPCF@SiNDs/C), shown in Fig. S27. When it was tested under the same condition as the MPCF@VG@SiNDs/C electrode, the rate capability (572.7 mAh g^−1^ at 20 A g^−1^, MPCF@SiNDs/C, Fig. S28; 910.3 mAh g^−1^ at 20 A g^−1^, MPCF@VG@SiNDs/C, Fig. 3c and electrode kinetics (Fig. S29) of MPCF@SiNDs/C are far inferior to those of MPCF@VG@SiNDs/C (detailed discussion in Supporting information), which supports the important role of VG on Li-ion transport in MPCF@VG@SiNDs/C. Consequently, an advanced MPCF@VG@SiNDs/C anode with unique low-strain property and rapid Li-ion transport capability is obtained, as illustrated in the schematic diagram in Fig. 5k.

### Electrochemical Performance Characterizations of Full Cells in LIBs

**Fig. 6:**
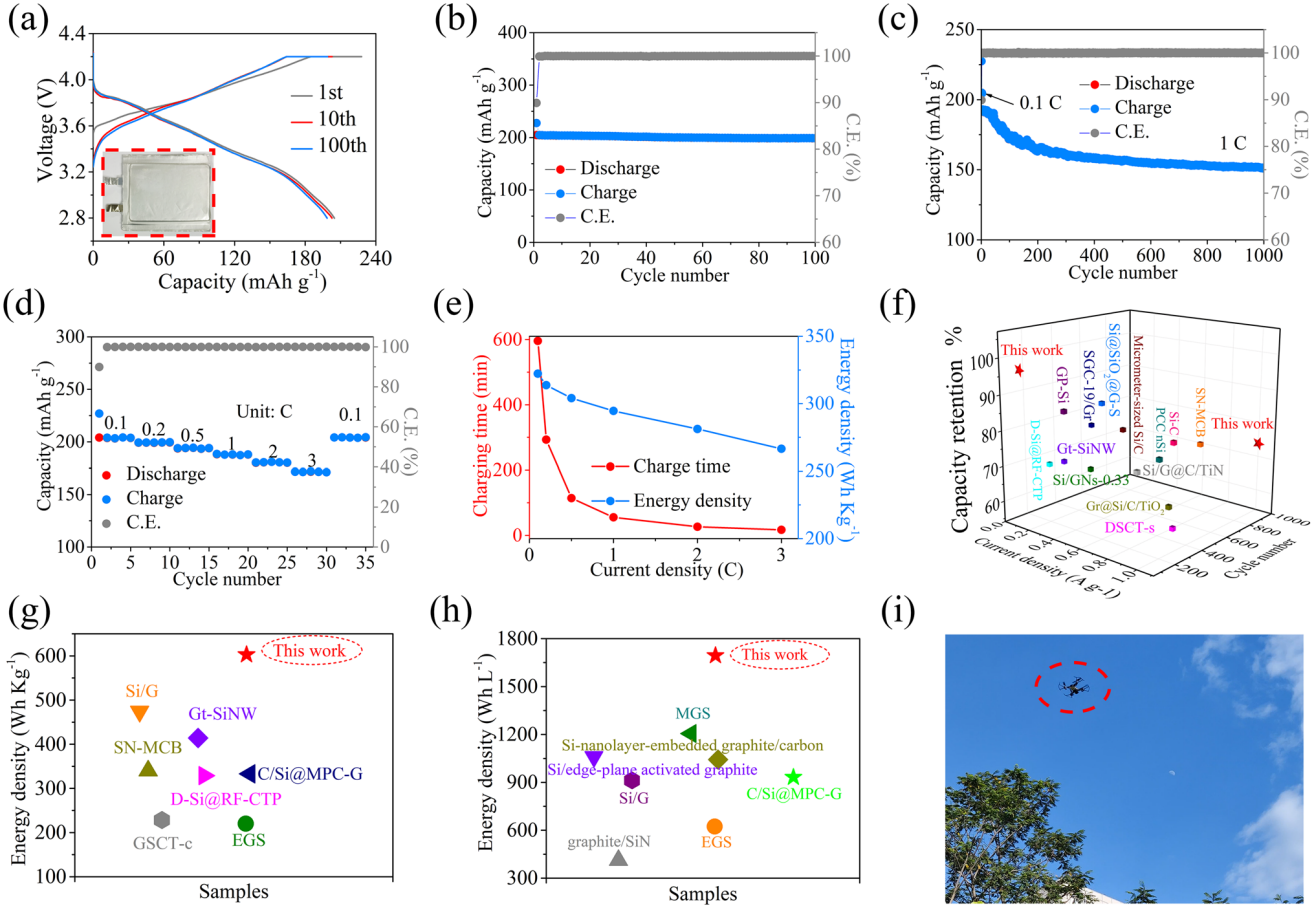
Full cells testing and performance comparison. **a** Galvanostatic charge/discharge voltage curves at 0.1 C (inset is the prepared pouch full cell), cycling performance at **b** 0.1 C and **c** 1 C, **d** rate performance, and **e** relationship between energy density and charging time of the pouch full cell. **f** Cycle stability, **g** gravimetric, and **h** volumetric energy densities comparison with previously reported silicon-carbon anodes. **i** Photographs of the drone in flight powered by the prepared pouch full cell

To assess the commercialization prospect of the MPCF@VG@SiNDs/C, the pouch full cells were prepared by using MPCF@VG@SiNDs/C as an anode and the commercial NCM811 as a cathode at an N/P ratio of 1.08. The purchased NCM811 cathode material displays a spherical morphology with a size of approximately 15 μm (Fig. S30a–c). It possesses the typical commercial NCM811 characteristics with a nominal voltage of ~ 3.77 V (Fig. S30d), a reversible specific capacity of 205.6 mAh g^−1^ (Fig. S30d, e), and rate capability (187.2 mAh g^−1^, 1 C; 170.7 mAh g^−1^, 3 C; Fig. S30f). The N/P ratio is an important parameter in battery design. In LIBs, an N/P ratio below 1 results in lithium plating on the anode surface, which compromises battery safety. Conversely, an excessively high N/P ratio leads to anode underutilization, reducing the battery’s energy density. Therefore, the N/P ratio for LIBs typically falls within the range of 1.05–1.1 [46, 47]. The pouch full cells are prepared and tested based on commercial full battery standards. The testing conditions of the pouch full cells are displayed in Table S7. In Fig. 6a, the prepared pouch full cell (inset in Fig. 6a) exhibits a discharging capacity of 204.7 mAh g^−1^ with an initial CE of 89.9%. The almost overlapping charge/discharge profiles of the 2nd and 100th cycles demonstrate the excellent stability of the electrode. The full cell delivers a high reversible capacity of 198.6 mAh g^−1^ with a capacity retention of 97.0% after 100 cycles at 0.1 C (Fig. 6b). After 1000 cycles at 1 C, the full cell still delivers a capacity of 151.0 mAh g^−1^ with a capacity retention of 78.3% (Fig. 6c), further denoting its excellent cycle stability. In Fig. 6d, the full cell delivers rate capacities of 203.7, 199.2, 193.8, 187.8, 180.4, and 171.5 mAh g^−1^ at 0.1, 0.2, 0.5, 1, 2, and 3 C, respectively. Significantly, the capacity retentions at 1 and 3 C are 92.2 and 84.2%, respectively, of that at 0.1 C, suggesting its excellent Li-ion transport capability. In addition, the gravimetric energy density of the pouch full cell is calculated to be 602.8 (according to the mass of active materials) and 322.2 Wh Kg^−1^ (according to the mass of active and inactive materials). Detailed calculation details are shown in Supporting Information. Notably, a high energy density of 498.5 Wh kg^−1^ (82.7% that at 0.1 C; according to active material) is obtained with a charging time of only 16.8 min at 3 C (Fig. 6e). Furthermore, the volumetric energy density of the full cell is up to 1694.0 (according to the active material thickness) and 1034.0 Wh L^−1^ (according to the active and inactive materials thickness). Clearly, the MPCF@VG@SiNDs/C has significant advantages in terms of fast-charging capability (Table S8), cycling stability (Fig. 6f and Table S9), gravimetric energy density (Fig. 6g, Tables S10 and S11), and volumetric energy density (Fig. 6h, Table S10) in the full cell compared to previously reported silicon-carbon anodes under industrial/non-industrial electrode standards. In particular, the fast-charging capability and cycling stability are, to the best of our knowledge, the highest level that has been reported. When the original cell of the drone is replaced with the prepared pouch full cell, the drone can fly normally, as shown in Figs. 6i, S31, and Video S1. These results fully demonstrate the great potential of MPCF@VG@SiNDs/C for practical use.

## Conclusions

In summary, a low-strain and fast-charging anode is designed by uniformly dispersing amorphous SiNDs in carbon nanospheres that are welded on the walls of MPCF by VG. The high dispersity and amorphous features of ultrasmall SiNDs (~ 0.7 nm) bring uniform stress distribution. The MPCF provides expansion space for SiNDs to eliminate the volume change of the whole MPCF@VG@SiNDs/C electrode to result in the low-strain property. VG as a bridge constructs directed and interconnected channels for rapid Li^+^/electron transport. As a result, the MPCF@VG@SiNDs/C shows a long cycling life (1000 cycles, 5 A g^−1^, 82.5% capacity retention), high capacity (1512.2 mAh g^−1^), and superior rate capability (909.3 mAh g^−1^ at 20 A g^−1^) in half cells. The pouch cell displays competitive gravimetric energy density (602.8 Wh kg^−1^), volumetric energy density (1694.0 Wh L^−1^), and fast-charging capability (498.6 Wh kg^−1^, charging time of 16.8 min at 3 C). This study offers a novel and effective strategy for the design and preparation of superior silicon-carbon anodes for practical energy applications.

## Supplementary Information

Below is the link to the electronic supplementary material.

Supplementary file1 (PDF 2823 KB)

Supplementary file2 (MP4 14757 KB)
