# Supplementary material for: Macroporous Directed and Interconnected Carbon Architectures Endow Amorphous Silicon Nanodots as Low-Strain and Fast-Charging Anode for Lithium-Ion Batteries
Source: Nanomicro Lett. 2024 Jan 29;16:98. doi: 10.1007/s40820-023-01308-x (PMC10825112; doi:10.1007/s40820-023-01308-x)
Supplement: Supplementary file 1 — Supplementary file1 (PDF 2823 KB) [file 40820_2023_1308_MOESM1_ESM.pdf]

Supporting Information for

Macroporous Directed and Interconnected Carbon Architectures Endow Amorphous Silicon Nanodots as Low-Strain and Fast-Charging Anode for Lithium-Ion Batteries

Zhenwei Li[1,2,#], Meisheng Han[3,#,*], Peilun Yu[1], Junsheng Lin[1], and Jie Yu[1,2,*]

[1] Guangdong Provincial Key Laboratory of Semiconductor Optoelectronic Materials and Intelligent Photonic Systems, Shenzhen Engineering Lab for Supercapacitor Materials, School of Material Science and Engineering, Harbin Institute of Technology, Shenzhen, University Town, Shenzhen 518055, P. R. China

[2] Songshan Lake Materials Laboratory Dongguan, Guangdong 523808, P. R. China

[3] Department of Mechanical and Energy Engineering, Southern University of Science and Technology, Shenzhen 518055, P. R. China

[#]Zhenwei Li and Meisheng Han contributed equally to this work.

*Corresponding authors. E-mail: jyu@hit.edu.cn (J. Yu); hanms@sustech.edu.cn (Meisheng Han)

S1 Electrochemical Performance Measurements

For the electrochemical characterization, the working electrodes were fabricated by mixing active materials, carbon black, styrene butadiene rubber, and sodium carboxymethyl cellulose at a mass ratio of 96:1:1.5:1.5 for MPCF@VG@SiNDs/C and 80:10:5:5 for SiNDs/C and VG@SiNDs/C, which was then uniformly coated on the copper foil and dried at 80 °C for 12 h under vacuum. Obviously, the slurry ratio in the preparation of MPCF@VG@SiNDs/C electrode is different from those of SiNDs/C and VG@SiNDs/C electrodes due to their different specific surface area. As shown in Fig. 2g, the specific surface areas of SiNDs/C and VG@SiNDs/C are far higher than that of MPCF@VG@SiNDs/C. Generally, larger specific surface areas need more binder to ensure the slurries coat on Cu foil. We once tried to prepare SiNDs/C and VG@SiNDs/C electrodes with the same binder as MPCF@VG@SiNDs/C, but it failed to form an electrode film. Besides, larger specific surface areas need more conductive agents to construct good electrode connectivity, thus ensuring the more fully reaction of Li^+ and active materials. We once tried to prepare SiNDs/C and VG@SiNDs/C electrodes with the same conductive agent as MPCF@VG@SiNDs/C, but their capacity and rate capability are poor, which does not make useful performance comparisons with MPCF@VG@SiNDs/C. Based on these results, we choose the different slurry ratios for these electrodes.

S2 Supplementary Figures and Tables

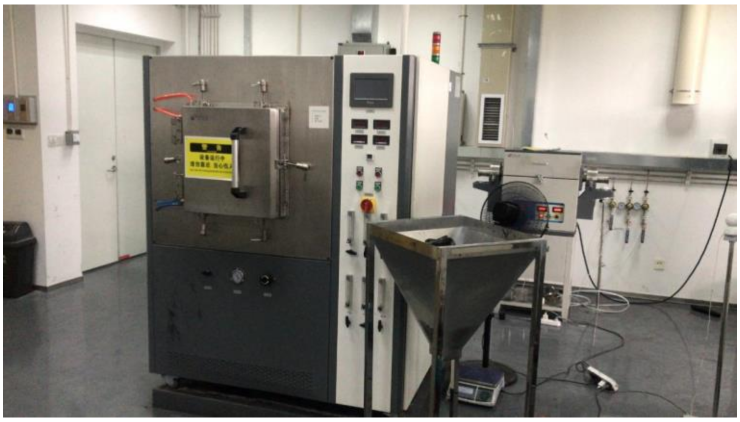

Fig. S1 Static sealed box furnace for the production of the SiNDs/C

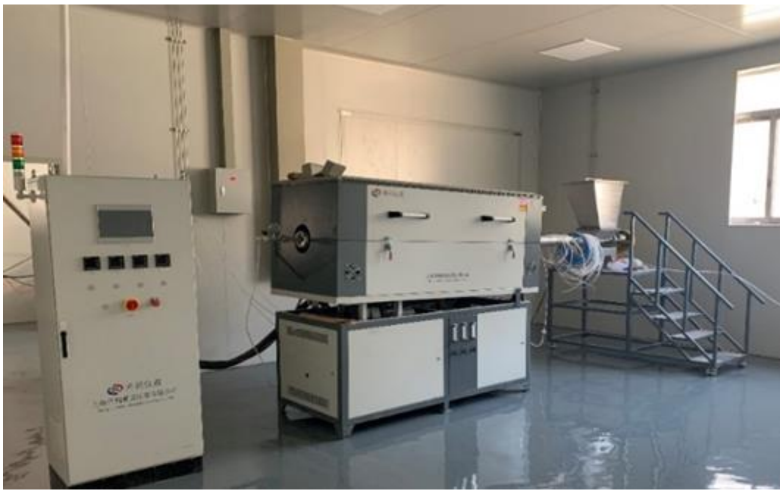

Fig. S2 Rotary sealed box furnace for growing VG on the SiNDs/C

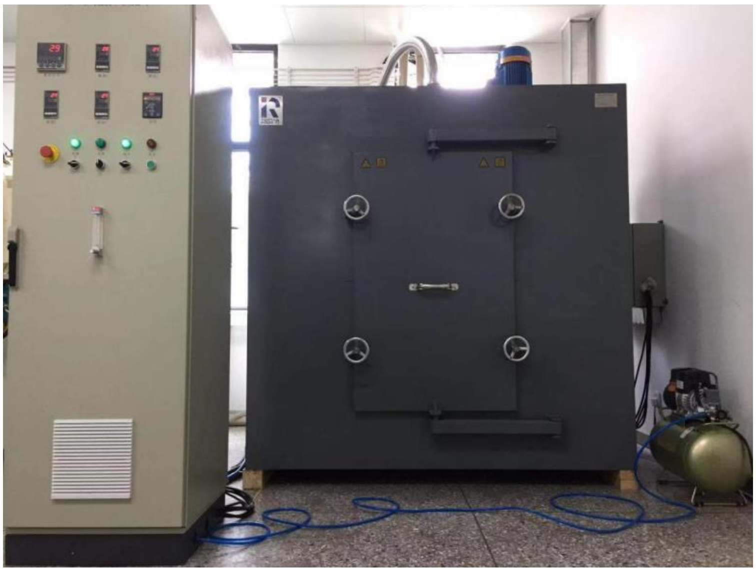

Fig. S3 Carbonization furnace for high-temperature carbonization

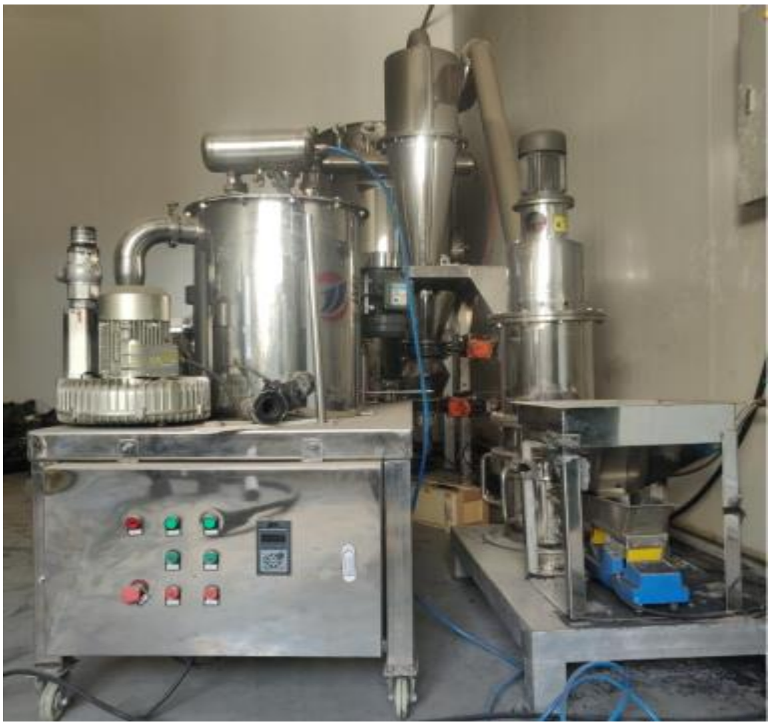

Fig. S4 Airflow crusher for sample crushing

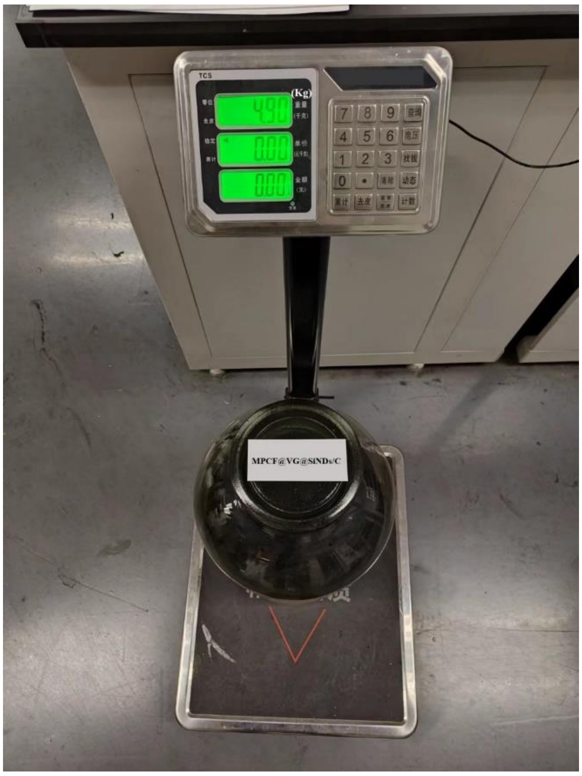

Fig. S5 Photos of the MPCF@VG@SiNDs/C material prepared by the kilogram.

The electronic platform scale has been peeled, i.e., the mass of the glass container has been deducted. That is, the total amount of MPCF@VG@SiNDs/C obtained after one preparations is 4.9 kg.

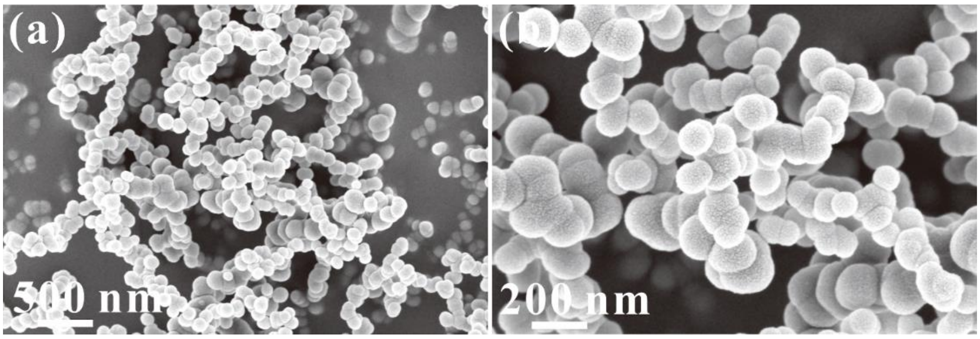

Fig. S6 SEM images of the SiNDs/C nanospheres

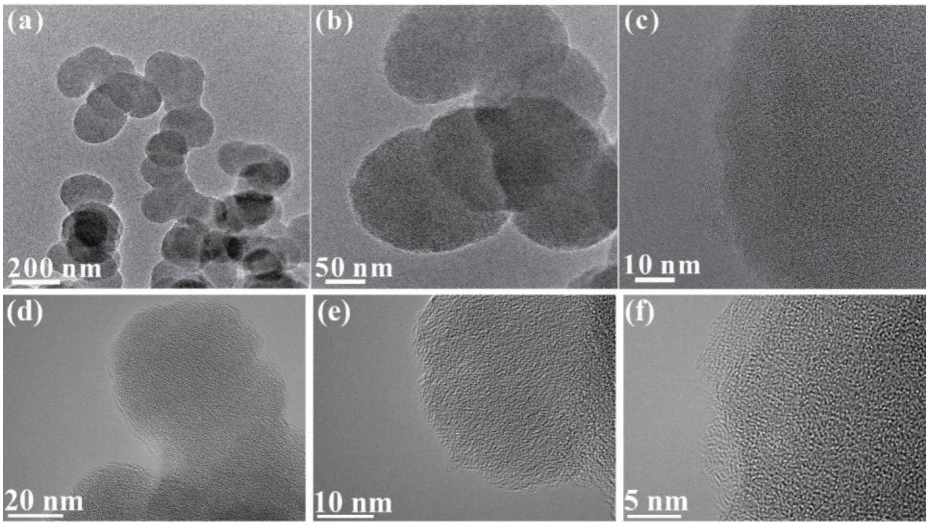

Fig. S7 (**a-c**) TEM and (**d-f**) AC-TEM images of the SiNDs/C nanospheres

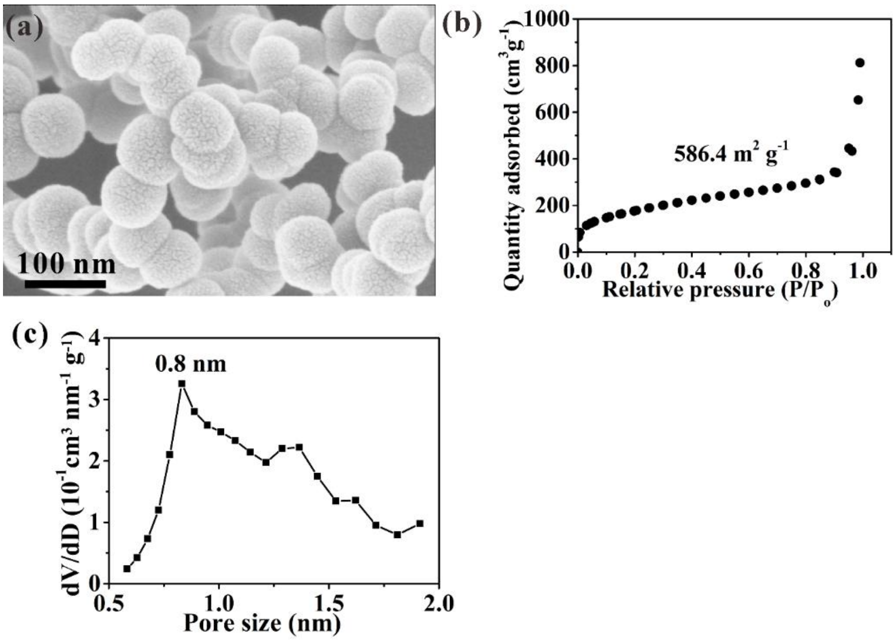

Fig. S8 a SEM image, **b** Nitrogen adsorption/desorption isotherms, and **c** pore size distribution of the SiNDs/C after NaOH etching

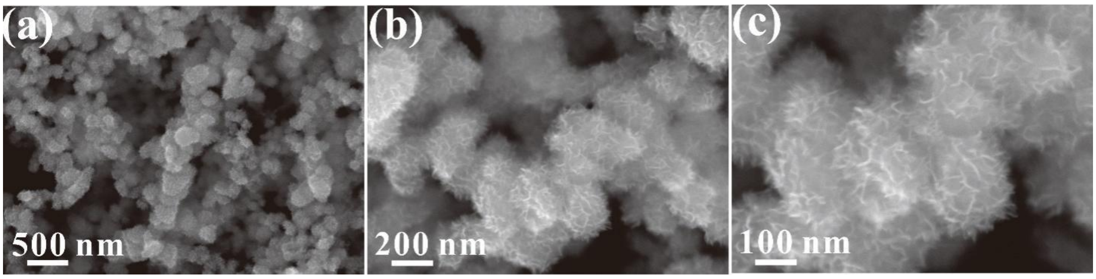

Fig. S9 SEM images of the VG@SiNDs/C

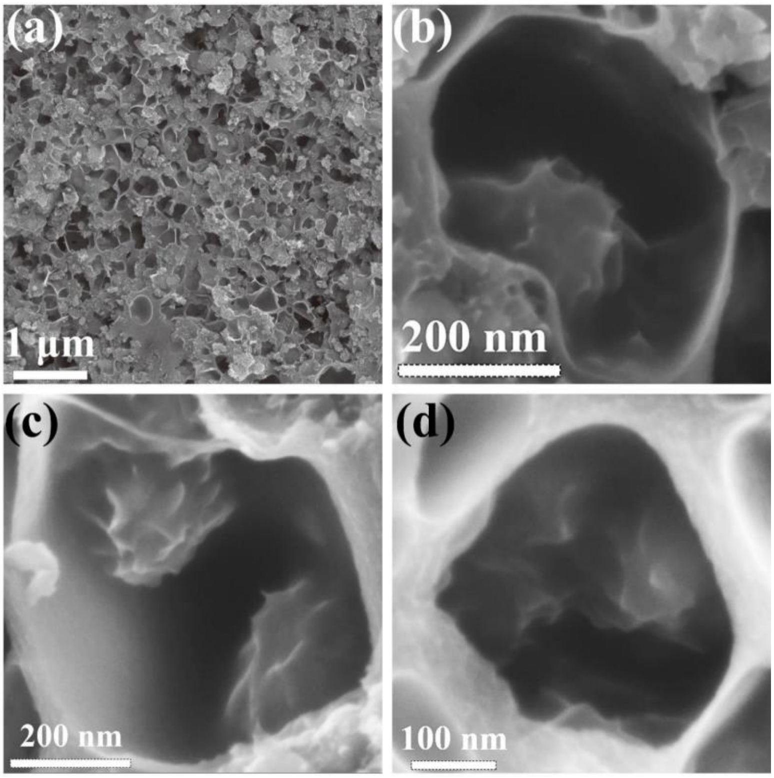

Fig. S10 SEM images of the MPCF@VG@SiNDs/C

Table S1 Particle size distribution (Unit: μm)

Samples	D_{min}	D_{10}	D_{50}	D_{90}	D_{max}
MPCF@VG@SiNDs/C	9.16	12.33	20.04	33.56	59.17

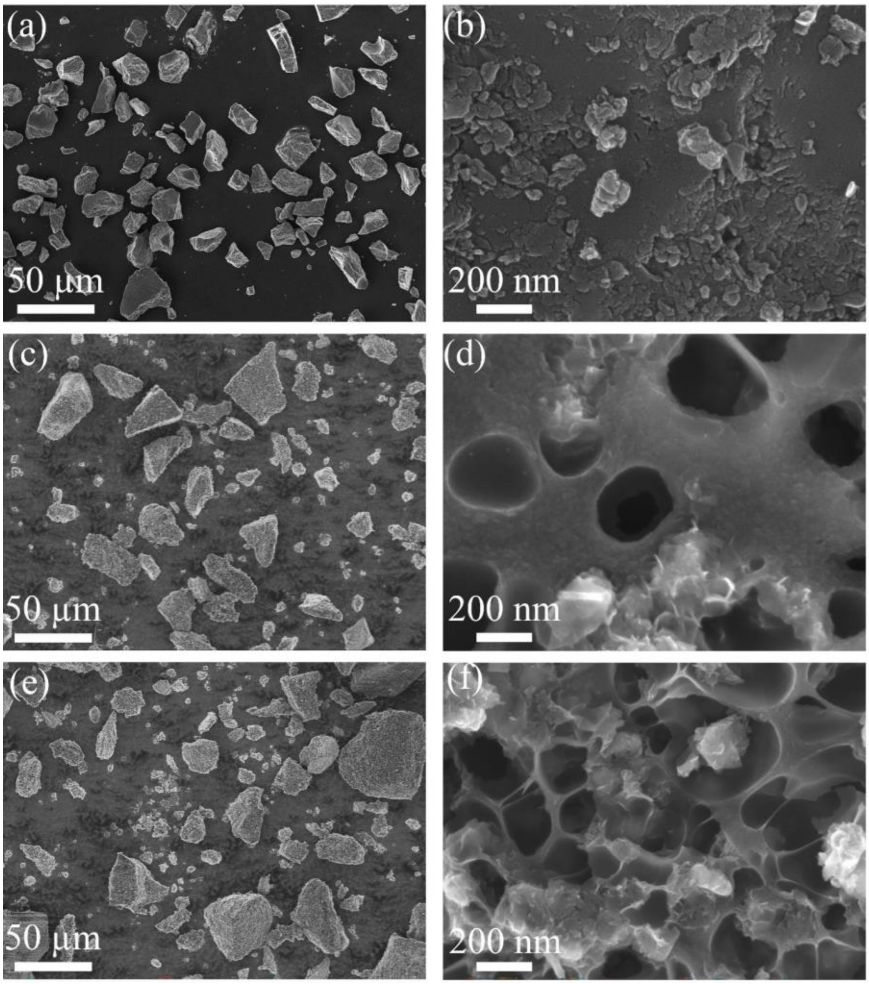

Fig. S11 SEM images of MPCF@VG@SiNDs/C with acetic acid/chitosan ratio of (**a, b**) 0 L/2.5 kg, (**c, d**) 2.5 L/2.5 kg, and (**e, f**) 7.5 L/2.5 kg during the granulatio

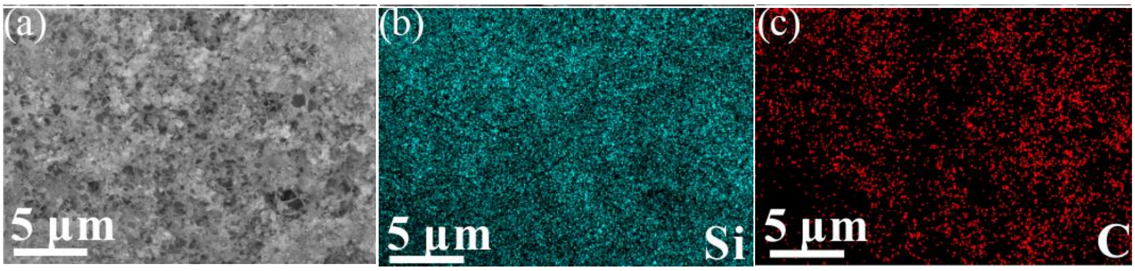

Fig. S12 **a** SEM image and **b, c** the corresponding EDS elemental mapping images of MPCF@VG@SiNDs/C

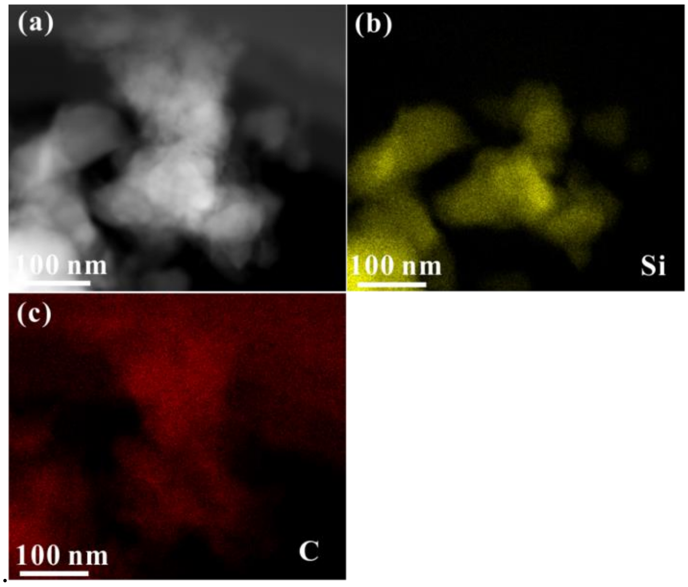

Fig. S13 **a** HAADF image and corresponding EDS images of **b** Si and **c** C of MPCF@VG@SiNDs/C. Before testing, the MPCF@VG@SiNDs/C is crushed with a mortar in order to reduce the size to observe

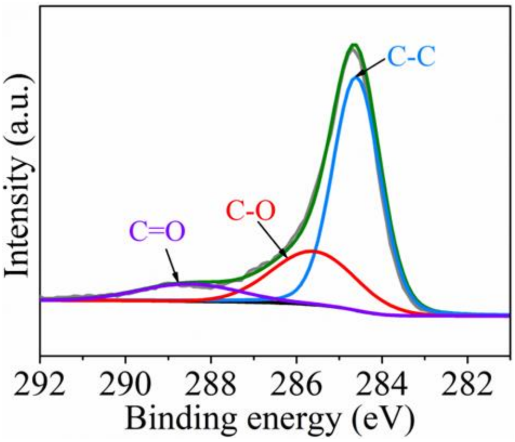

Fig. S14 High-resolution XPS spectra for C 1s for the SiNDs/C

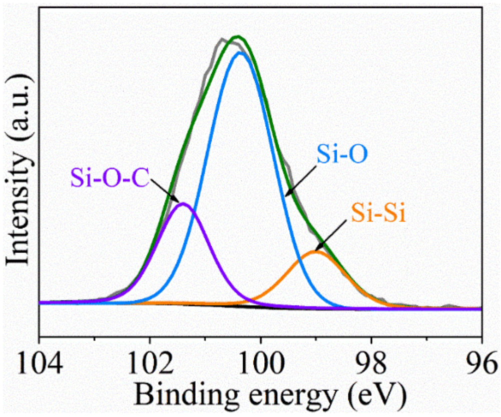

Fig. S15 High-resolution XPS spectrum for Si 2p for the pyrolysis product at 1000 °C under Ar atmosphere without H_2 reduction

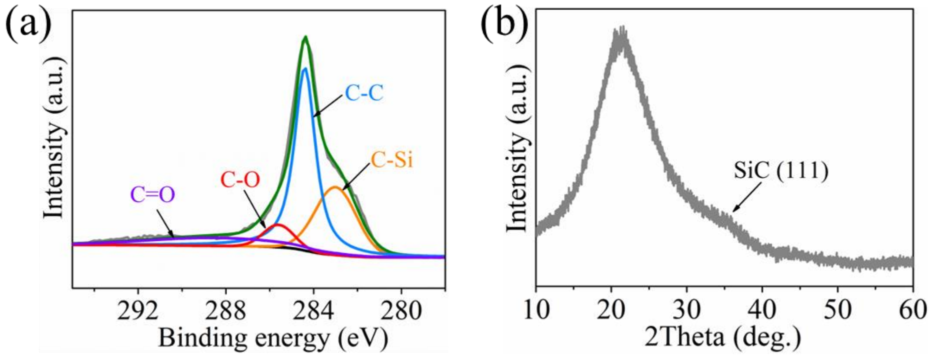

Fig. S16 a High-resolution XPS spectrum for C 1s and **b** XRD pattern of the pyrolysis product at 1000 °C with an OMCTS/H_2 flow ratio of 1/8

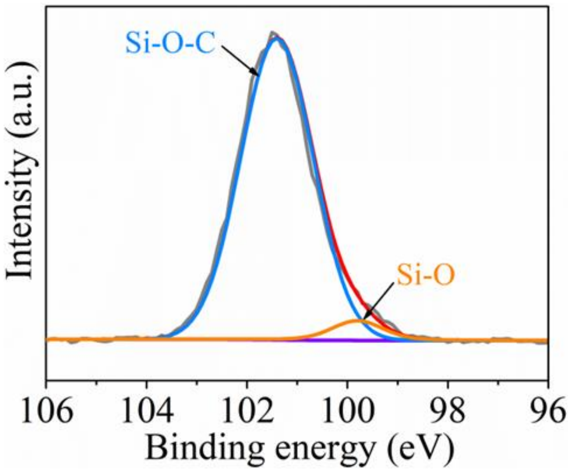

Fig. S17 High-resolution XPS spectrum for Si 2p of the pyrolysis product at 800 °C with an OMCTS/H_2 flow ratio of 1/4

Table S2 The tap density and electrode compaction density of the obtained samples

Samples	Tap density (g cm^{-3})	Electrode compaction density (g cm^{-3})
SiNDs/C	0.24	0.44
VG@SiNDs/C	0.22	0.47
MPCF@VG@SiNDs/C	0.82	1.38

Table S3 The first CE (%), reversible capacity (mAh g^{-1}), and capacity retention (%) after 100 cycles of the obtained samples

Samples	First CE	First capacity	Final capacity	Capacity retention
SiNDs/C	78.1	2134.6	1908.1	89.4
VG@SiNDs/C	71.7	1794.6	1716.8	95.7
MPCF@VG@SiNDs/C	88.6	1512.2	1541.0	102.0

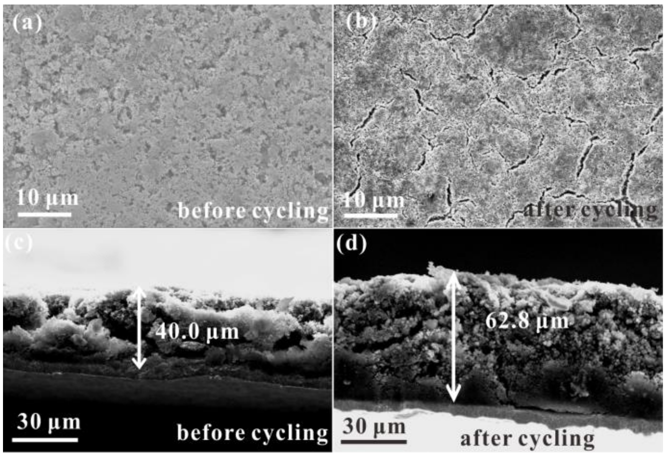

Fig. S18 Top-view SEM images of the SiNDs/C electrodes **a** before and **b** after 100 cycles at 0.1 A g^{-1}. Side-view SEM images of the SiNDs/C electrodes **c** before and **d** after 100 cycles at 0.1 A g^{-1}

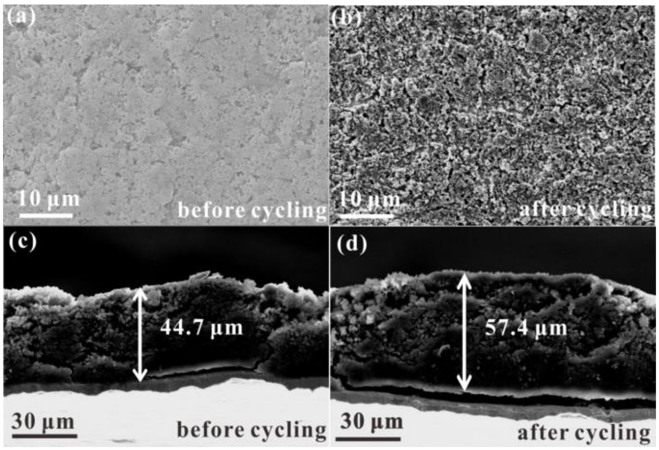

Fig. S19 Top-view SEM images of the VG@SiNDs/C electrodes **a** before and **b** after 100 cycles at 0.1 A g^{-1}. Side-view SEM images of the VG@SiNDs/C electrodes **c** before and **d** after 100 cycles at 0.1 A g^{-1}

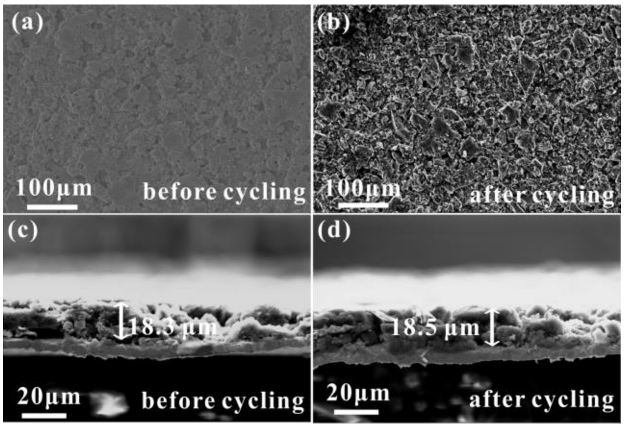

Fig. S20 Top-view SEM images of the MPCF@VG@SiNDs/C electrodes **a** before and **b** after 100 cycles at 0.1 A g^{-1}. Side-view SEM images of the MPCF@VG@SiNDs/C electrodes **c** before and **d** after 100 cycles at 0.1 A g^{-1}

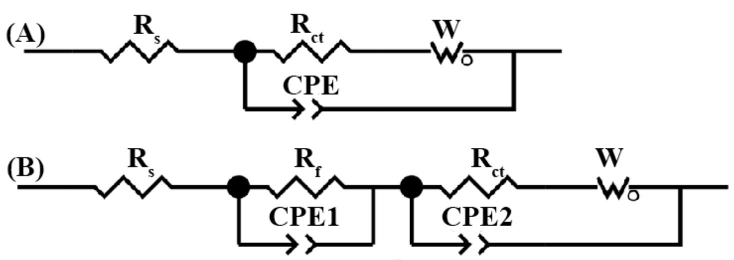

Fig. S21 The fitted circuits of EIS spectra in Figs. 3g-i. **a** before cycling, **b** after cycling

Table S4 R_s, R_{ct}, R_f, and the slop of the sloping line of electrodes in low-frequency region before cycling and after cycling. The data are from the fitted circuit of EIS spectra in the manuscript in Figs. 3g-i

Samples	R_s	R_{ct}	R_f	slope
SiNDs/C, before cycling	2.5	122.6	0	2.0
VG@SiNDs/C, before cycling	2.2	91.4	0	3.9
MPCF@VG@SiNDs/C, before cycling	1.7	56.2	0	7.5
SiNDs/C, after the 1st cycling	3.9	76.9	13.8	1.3
VG@SiNDs/C, after the 1st cycling	2.7	47.4	7.4	1.8
MPCF@VG@SiNDs/C, after the 1st cycling	2.0	33.4	5.2	4.0
SiNDs/C, after testing rate cycling	5.5	33.6	15.6	1.0
VG@SiNDs/C, after testing rate cycling	3.5	20.1	8.1	1.6
MPCF@VG@SiNDs/C, after testing rate cycling	2.5	13.2	5.7	3.3

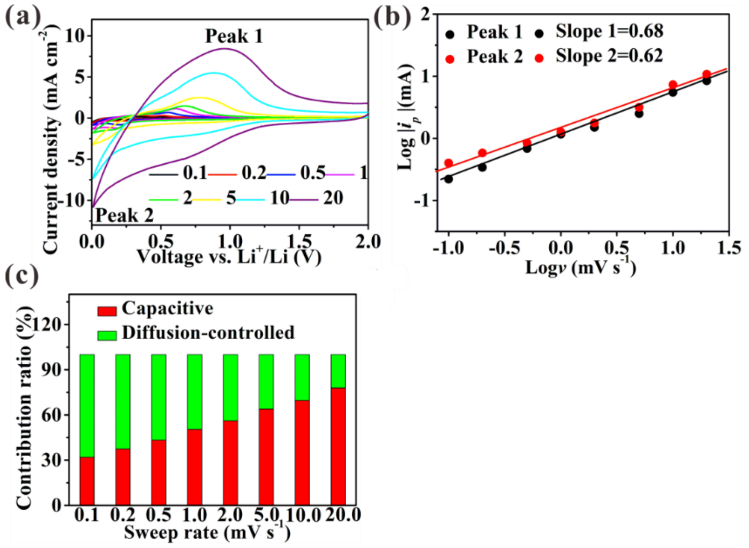

Fig. S22 The electrochemical kinetic analysis of SiNDs/C: **a** CV curves at different sweep rates, **b** Log i_p against Log v at marked peaks, **c** The percentages of pseudocapacitive contribution at different sweep rates

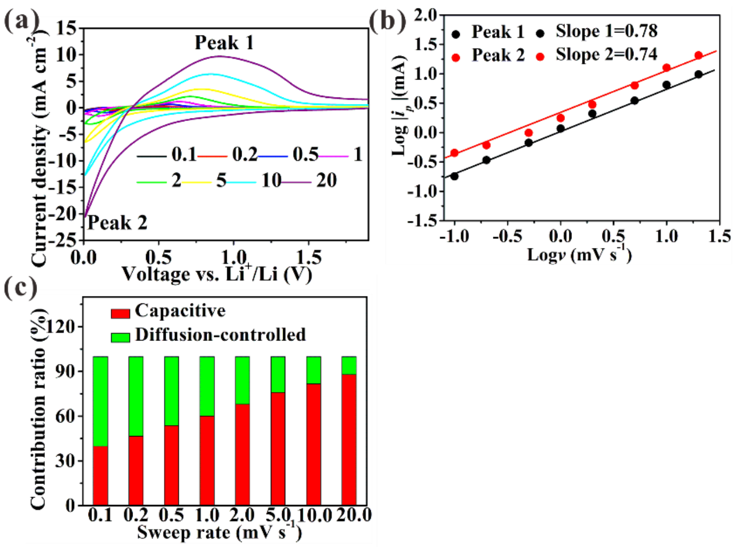

Fig. S23 The electrochemical kinetic analysis of VG@SiNDs/C: **a** CV curves at different sweep rates, **b** Log i_p against Log v at marked peaks, **c** The percentages of pseudocapacitive contribution at different sweep rates

Table S5 Comparison of electrochemical performances of MPCF@VG@SiNDs/C with previously reported Si/C composites for LIBs anodes **under industrial electrode conditions** in open reports. C_C-final charge capacity (mAh g^{-1}), ICE-initial Coulombic efficiency (%), C_R-capacity retention (%), M_L-mass loading (mg cm^{-2}), Ac-areal capacity (mAh cm^{-2}), J-current density (A g^{-1}), N_C-cycle number, NA-not available

Samples	C_C	ICE	C_R	M_L	Ac	J	N_C	References
**MPCF@VG@SiNDs/C**	**1541.0**	**88.6**	**102.0**	**2.52**	**3.81**	**0.1**	**100**	**This work**
**MPCF@VG@SiNDs/C**	**1301.4**	**88.3**	**96.3**	**2.52**	**3.81**	**1**	**1000**	**This work**
**MPCF@VG@SiNDs/C**	**975.6**	**88.0**	**82.6**	**2.52**	**3.81**	**5**	**1000**	**This work**
SN-MCB	~1500	~80	80	1.5	2.7	0.8	500	Nano Lett. 2019, 20, 625-635
CNT@Si@C	1595.7	75	90	1.88	3.0	~ 0.4	100	Nature Commun. 2020,11, 1-9
CNT@Si@C-Gr	718	~84	92	4	3.0	~ 0.2	500	Nature Commun. 2020,11, 1-9
SGC_{pitch}	523	90.9	96.9	7	3.9	0.5	50	Adv. Energy Mater. 2019, 9, 1803121
C/Si@MPC-G	665	90.9	95.6	/	3.6	0.5	50	Adv. Mater. 2020, 32, 2003286
Gt-SiNW	644	~77	87	2.7	3.0	2	250	ACS Nano 2020, 14, 12006-12015
SGC	525	92	96	/	3.3	0.5	100	Nat. Energy, 2016, 1, 16113

Table S6 Comparison of electrochemical performances of MPCF@VG@SiNDs/C with previously reported Si/C composites for LIBs anodes **under non-industrial electrode conditions** in open reports. C_{R1}-capacity retention (%) in cycling performances, C_{R2}-capacity retention (%) relative to capacity obtained at the lowest current density in rate performances, M_L-mass loading (mg cm^{-2}), Ac-areal capacity (mAh cm^{-2}) calculated at low current density, J-current density (A g^{-1}), N_C-cycle number, NA-not available.

Samples	C_{R1}	C_{R2}	M_L	Ac	J	N_C	References
**MPCF@VG@SiNDs/C**	**102.0**	/	**2.52**	**3.81**	**0.1**	**100**	**This work**
**MPCF@VG@SiNDs/C**	**96.3**	**89.2**	**2.52**	**3.81**	**1**	**1000**	**This work**
**MPCF@VG@SiNDs/C**	**82.5**	**78.9**	**2.52**	**3.81**	**5**	**1000**	**This work**
**MPCF@VG@SiNDs/C**	/	**60.2**	**2.52**	**3.81**	**20**	**NA**	**This work**
Si/C-CVD	~ 85	NA	1.25	NA	0.1	100	Energy Stor. Mater. 2023, 102857
Si/C-SD	~ 64	NA	1.25	NA	0.1	100	Energy Stor. Mater. 2023, 102857
Si/SiO_2@Graphene	~ 94	NA	0.6		1	130	Adv. Funct. Mater. 2023, 33, 2211648
Si/SiO_2@Graphene	~ 93	NA	0.6		4	130	Adv. Funct. Mater. 2023, 33, 2211648
Si/SiO_2@Graphene	~ 78	NA	0.6		2	500	Adv. Funct. Mater. 2023, 33, 2211648
Si/SiO_2@Graphene	~ 80	NA	3		0.4	80	Adv. Funct. Mater. 2023, 33, 2211648
D-Si@RF-CTP	80.3	NA	0.7	0.8	0.5	200	Energy Stor. Mater. 2023,56, 40-49
GSCT-c	~ 80	NA	1.07		1.2	200	Energy Stor. Mater. 2023,56, 319-330
GSCT-c	~ 50	NA	3.04		1.2	200	Energy Stor. Mater. 2023,56, 319-330
Gr@Si/C/TiO_2	~ 70	NA	1.2		1.2	1000	J. Energ. Chem. 2023, 77, 348-358
Si NDs⊂MDN	90.1	83.1	2	~2.9	1	1000	Adv. Mater. 2022, 34, 2200894
PCSi-2	62.8	NA	~1	~2	1	500	Adv. Mater. 2022, 34, 2109658
PCSi-2	NA	20.5	~1	~2	16.5	NA	Adv. Mater. 2022, 34, 2109658

Si@25-PU	~50		0.9	~2.6	0.8	1000	Nano Energy 2022, 103, 107829
Si-NSs@rGO	50	~85	NA	NA	2	1000	Adv. Funct. Mater. 2022, 32, 2110046
Si-NSs@rGO	NA	70	NA	NA	10	NA	Adv. Funct. Mater. 2022, 32, 2110046
HD-Si@$Ti_3C_2T_x$@G	63.1	37.8	1-2	~3.4	1	800	ACS Nano 2022, 16, 4642-4653
Si nanotubes	53	55.8	1	~2.9	1	1000	ACS Nano 2022, 16, 7689-7700
PCC nSi	83.7	~74	1.3	2.6	1	1000	Energy Stor. Mater. 2021, 34, 768-777
Si@C-Ni	~55	NA	NA	NA	0.2	1000	Energy Stor. Mater. 2021, 39, 1-10
Sip-NS@TNSs	48	NA	1	~2.2	1	2000	ACS Nano 2020, 14, 5111-5120
SHCM/NCF	86	NA	1	~2	1	800	Adv. Funct. Mater. 2021, 31, 2101487
SGC	83.3	~60	NA	NA	2	380	Energy Stor. Mater. 31 (2020) 36-43
SGC	NA	32.1	NA	NA	20	NA	Energy Stor. Mater. 31 (2020) 36-43
Si@void@C	73.5	59.1	1	~2.4	2	1000	ACS Nano 2019, 13, 12219-12229

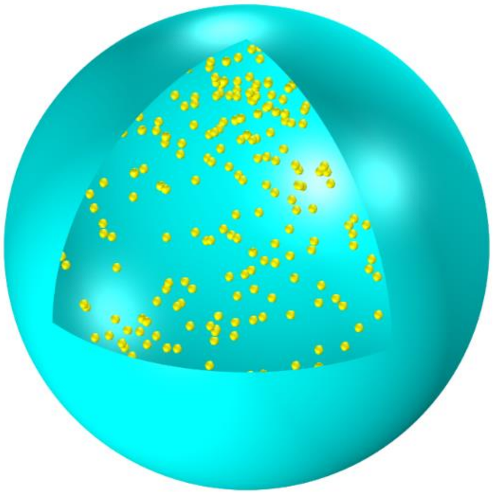

Fig. S24 The finite element model established for uniform distribution of crystalline/amorphous Si nanodots in amorphous carbon nanospheres

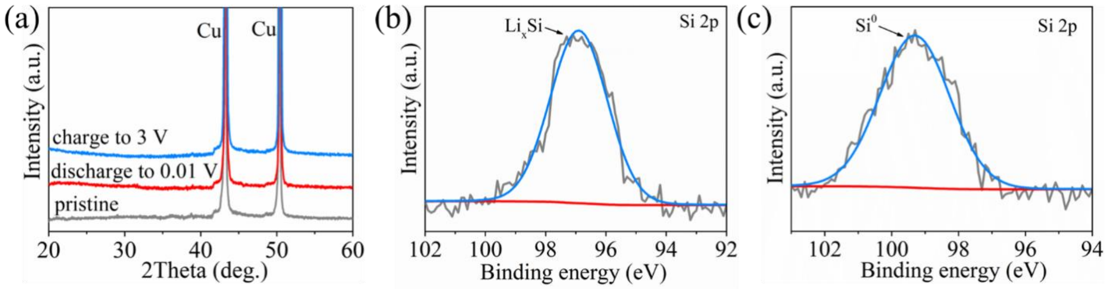

Fig. S25 a XRD patterns of SiNDs/C before and after the charge/discharge process. High-resolution XPS Si 2p spectra of SiNDs/C after **b** discharging to 0.01 V and **c** charging to 3 V

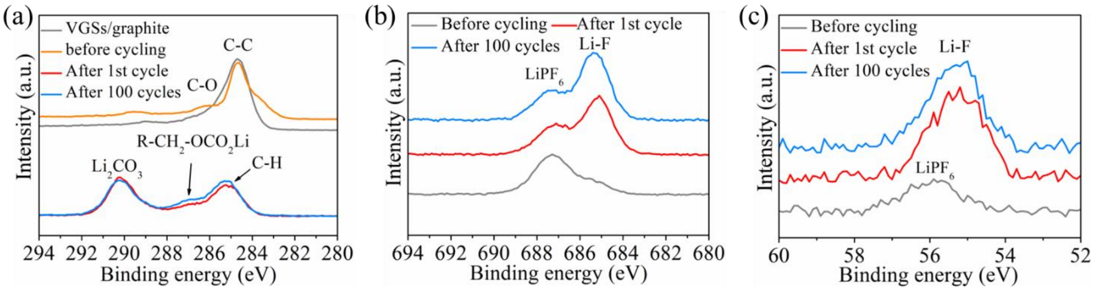

Fig. S26 XPS spectra of MPCF@VG@SiNDs/C before and after cycling. **a** C 1s, **b** F 1s, and **c** Li 1s

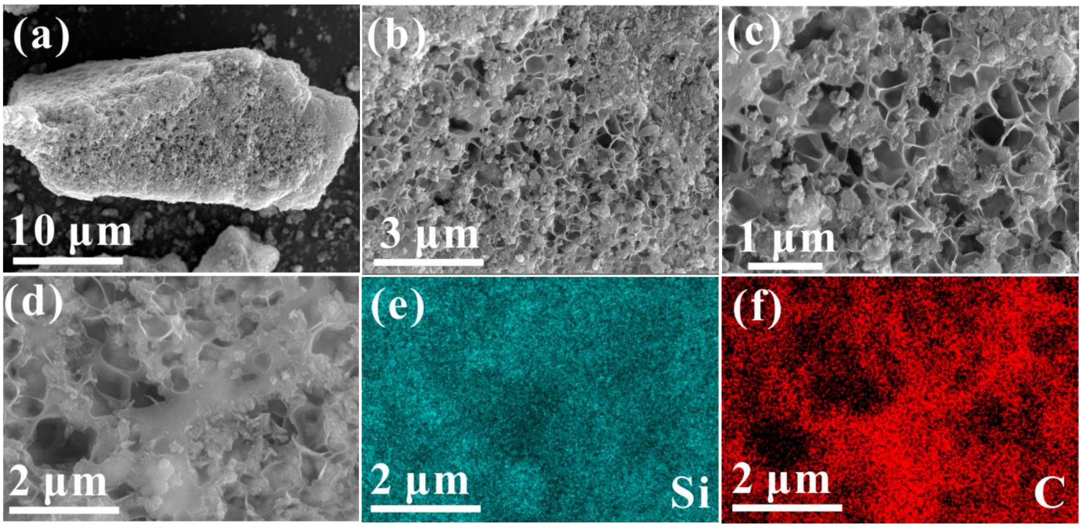

Fig. S27 SEM images **a-c** and corresponding EDS images **d-f** of MPCF@SiNDs/C

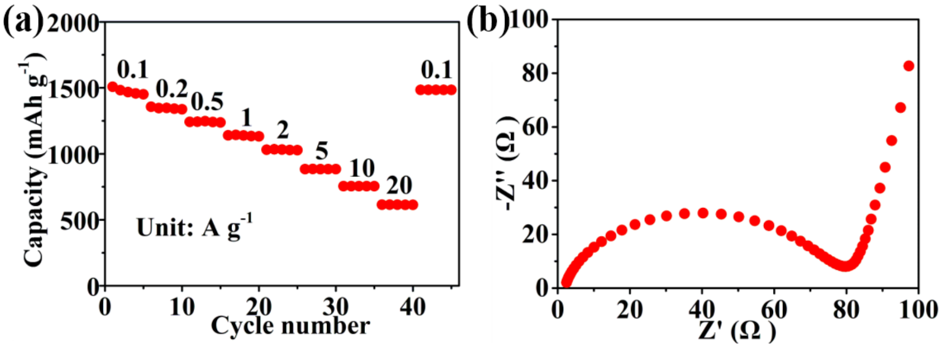

Fig. S28 a Rate curve and **b** EIS spectra of MPCF@SiNDs/C

According to Fig. S28a, the rate capacities of MPCF@SiNDs/C at 0.1, 0.2, 0.5, 1, 2, 5, 10, and 20 A g^{-1} are 1430.6, 1321.4, 1252.7, 1183.6, 1054.1, 872.2, 756.1, and 572.7 mAh g^{-1}, respectively. By comparison with the rate performance of MPCF@VG@SiNDs/C in Fig. 3c, it can be concluded that the rate capacities of MPCF@VG@SiNDs/C are much higher than MPCF@SiNDs/C, demonstrating the important role of VG on Li-ion transport. Before cycling (Fig. S28b), the value of R_{ct} of MPCF@SiNDs/C is larger than MPCF@VG@SiNDs/C, further confirming the significant advantages of MPCF@VG@SiNDs/C on charge transport capability.

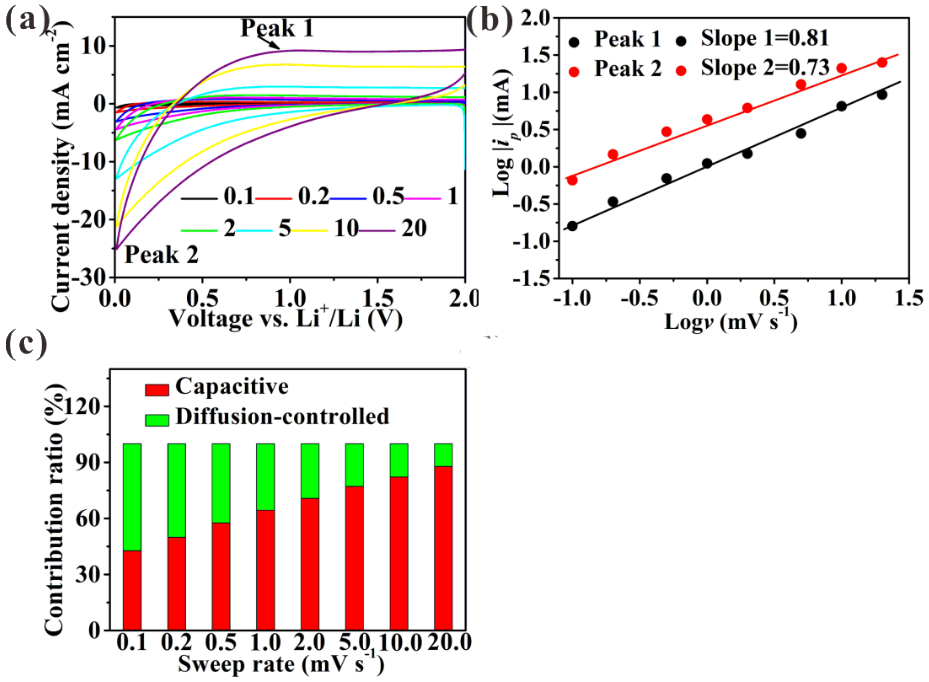

Fig. S29 The electrochemical kinetic analysis of MPCF@SiNDs/C: **a** CV curves at different sweep rates, **b** Log i_p against Log v at marked peaks, **c** The percentages of pseudocapacitive contribution at different sweep rates

To compare the Li^+ storage kinetics in MPCF@SiNDs/C and MPCF@VG@SiNDs/C electrodes, we perform CV measurements on MPCF@SiNDs/C electrode with sweeping rates ranging from 0.1 to 20 mV s^{-1} (Fig.

S29a). For MPCF@SiNDs/C, *b* values are calculated to be 0.81 and 0.73 for peaks 1 and 2, respectively (Fig. S29b), which are lower than those of MPCF@VG@SiNDs/C electrode (*b* values are calculated to be 0.91 and 0.84 for peaks 1 and 2, respectively, Fig. 4e). This result demonstrates that the MPCF@VG@SiNDs/C electrode has a higher capacitive contribution on capacity compared to MPCF@SiNDs/C electrode. As the scan rate increases from 0.1 to 20 mV s^{-1}, the contribution of capacitance rises from 44.1% to 82.6% for the MPCF@SiNDs/C electrode (Fig. S29c), which is much lower than MPCF@VG@SiNDs/C electrode (46.9 to 96.5%, Fig. 4f). The higher capacitive contribution is beneficial for obtaining higher capacity and better rate capability. These results indicate that MPCF@VG@SiNDs/C has a significant advantage over MPCF@SiNDs/C in promoting Li-ion transport.

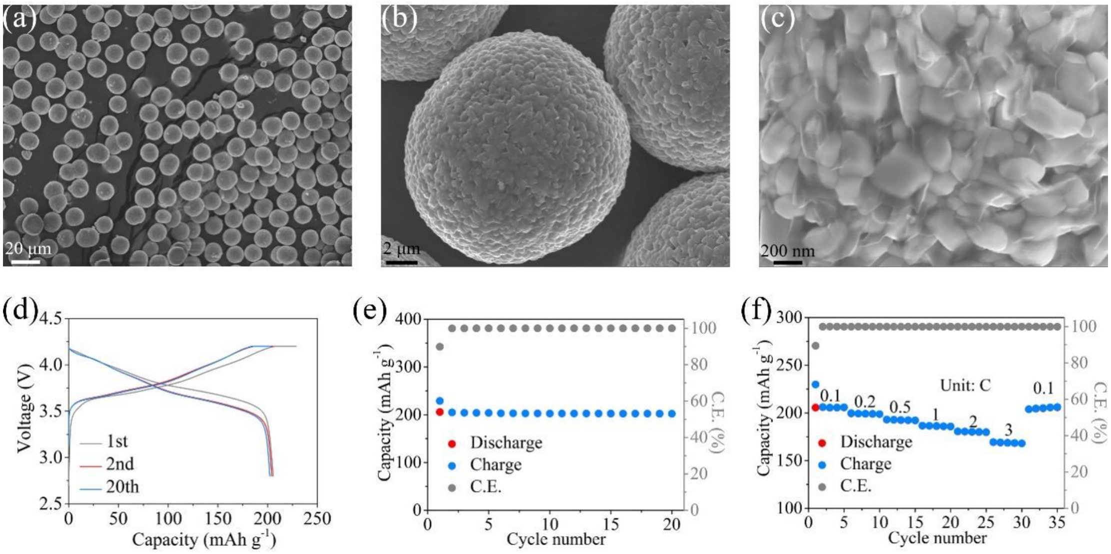

Fig. S30 a, b, c SEM images of the NCM811. **d** Galvanostatic charge/discharge voltage curves at 0.1 C, **e** cycling performance at 0.1 C, and **f** rate performance of the NCM811

Table S7 The specific testing conditions in full cell. The full cells are charged to 4.2 V at a current density; afterward, a constant voltage is applied at 4.2 V with a cut-off relatively low current density. Then, after 5 min quiescence, the full cells are discharged to 2.8 V at a current density

Testing current density	Charging (Constant current)	Charging (Constant voltage)	Discharging (Constant current)
0.1 C	0.1 C	0.01 C	0.1 C
0.2 C	0.2 C	0.01 C	0.2 C
0.5 C	0.5 C	0.01 C	0.3 C
1.0 C	1.0 C	0.02 C	0.6 C
2.0 C	2.0 C	0.02 C	1.2 C
3.0 C	3.0 C	0.02 C	1.8 C

The gravimetric energy density can be obtained according to Eq. S1 [S1, S2].

Gravimetric energy density (Wh kg^{-1}) $= \left(\frac{Cc \times V}{(m_{active} + m_{inactive})} \right)$ (S1)

Where V, C_c, m_{active}, and $m_{inactive}$ represent the nominal voltage (3.46 V), cell capacity (2 Ah), active mass (cathode and anode, 11.48 g), and inactive mass (conductive agent, electrolyte, binder, separator, current collectors, external packing material, 10.00 g), respectively.

The volumetric energy density of the full cell obeys Eq. S2 [S3].

Volumetric energy density (Wh L^{-1}) $= \left(\frac{Cc \times V}{(t_{active} + t_{inactive})} \right)$ (S2)

Where V, C_c, t_{active}, and $t_{inactive}$ represent the nominal voltage (3.46 V), cell capacity (89.7 mAh cm^{-2}), the thickness of active mass (cathode and anode, 0.183 cm), and inactive mass (separator, and current collectors, 0.117 cm), respectively.

Table S8 Fast-charging performances of various materials in full cells. C_R-Capacity retention (%) compared with the pristine value obtained at 0.1 C, Cycle number-C_N, J-current density (C)

Samples	C_R	C_N	J	References
**MPCF@VG@SiNDs/C**	**92.2**	**25**	**1 C**	**This work**
**MPCF@VG@SiNDs/C**	**88.6**	**30**	**2 C**	**This work**
**MPCF@VG@SiNDs/C**	**84.2**	**35**	**3 C**	**This work**
Si/SiO_2@G-S	~ 67	10	3 C	Adv. Funct. Mater. 2023, 33, 2211648
Si (7 μm)-WMG_H	56.8	20	2 C	Energy Stor. Mater. 2022, 50, 234-242
Si/G@C/TiN	77.0	25	2 C	Energy Stor. Mater. 2020, 29, 367-376
Si-C	73.0	15	3 C	Nano Lett. 2020, 20, 625-635.
C/Si/graphite	48.6	25	3 C	J. Power Sources 2020, 457, 228021.
SiO_x/C	89.7	20	1 C	Energy Stor. Mater. 2018, 112-118.
Si/edge-plane activated graphite	~80.0	15	3 C	Nat. Commun. 2017, 8, 812.
Silicon-nanolayer-embedded graphite/carbon	88.0	20	1 C	Nat. Energy 2016, 1, 16113.

Table S9 Comparison of cycling stability of various materials under high current densities in full cells. C_R-Capacity retention (%), Cycle mumber-N_C, J-current density (C)

Samples	C_R	N_C	J	References
**MPCF@VG@SiNDs/C**	**97.0**	**100**	**0.1**	**This work**
**MPCF@VG@SiNDs/C**	**78.3**	**1000**	**1**	**This work**
D-Si@RF-CTP	70.6	200	0.5	Energy Stor. Mater. 2023,56, 40-49
GSCT-c	60.4	300	1	Energy Stor. Mater. 2023,56, 319-330
Gr@Si/C/TiO_2	66.7	270	1	J. Energ. Chem. 2023, 77, 348-358
Si/SiO_2@G-S	~89	300	0.5	Adv. Funct. Mater. 2023, 33, 2211648
GP-Si	89.6	50	0.5	Adv. Funct. Mater. 2022, 32, 2107897
Micrometer-sized Si/C	80	450	0.5	Adv. Mater. 2021, 33, 2103095
Si/GNs-0.33	67.4	400	0.3	Energy Stor. Mater. 2021, 34, 311-319
PCC nSi	80.0	200	1	Energy Stor. Mater. 2021, 34, 768-777
SGC-19/Gr	78.1	500	0.2	Energy Stor. Mater. 2021, 35, 317-326
SN-MCB	80	500	1	Nano lett. 2020, 20, 625-635
Gt−SiNW	70	300	0.2	ACS Nano 2020, 14, 12006-12015
Si-C	83	300	1	Nano Energy 2020, 76, 105065
Si/G@C/TiN	79.0	50	1	Energy Stor. Mater. 2020, 29, 367-376

Table S10 Comparison of E_V and E_G of our full cells with previosuly reported full cells when Si/C composites as anode materials in LIBs **based on industrial electrode conditions**

Samples	E_V, (Wh L^{-1})	E_G, (Wh kg^{-1})	References
**MPCF@VG@SiNDs/C**	**1694.0**	**602.8**	**This work**
D-Si@RF-CTP	/	329	Energy Stor. Mater. 2023,56, 40-49
GSCT-c	/	288.4	Energy Stor. Mater. 2023,56, 319-330
EGS	623	220	Adv. Energy Mater. 2020, 10, 1903400
graphite/SiN	412.2	/	Energy Environ. Sci. 2020, 13, 1212-1221.
SN-MCB	/	340	Nano lett. 2020, 20, 625-635
Si/G	912	474	Energy Environ. Sci. 2020, 13, 3723-3731
Gt-SiNW	/	414	ACS Nano 2020, 14, 12006-12015
C/Si@MPC-G	932	333	Adv. Mater. 2020, 32, 2003286
MGS	1205.6	/	Nat. Commun. 2019, 10, 1-10
Si/edge-plane activated graphite	1060	/	Nat. Commun. 2017, 8, 812.
Silicon-nanolayer-embedded graphite/carbon	1043	474	Nat. Energy 2016, 1, 16113.

Table S11 Comparison of E_V and E_G of our full cells with previosuly reported full cells when Si/C composites as anode materials in LIBs based on **non-industrial electrode conditions**

Samples	E_V, (Wh L^{-1})	E_G, (Wh kg^{-1})	References
**MPCF@VG@SiNDs/C**	**1694.0**	**602.8**	**This work**
Gr@Si/C/TiO_2	/	489.3	J. Energ. Chem. 2023, 77, 348-358
Si NDs⊂MDN	/	366	Adv. Mater. 2022, 34, 2200894
p-Si@C	/	361.6	Energy Stor. Mater. 2022, 46, 384-393
Si-Gr	/	390	Energy Stor. Mater. 2022, 49, 111-121
Si@25-PU	/	453	Nano Energy 2022, 103, 107829
PCC nSi	/	466	Energy Stor. Mater. 2021, 34, 768-777
Si p-NS@TNSs	/	405	ACS Nano 2020, 14, 5111-5120
Gt−SiNW	/	414	ACS Nano 2020, 14, 12006-12015
Si/G@C/TiN	/	476	Energy Stor. Mater. 2020, 29, 367-376

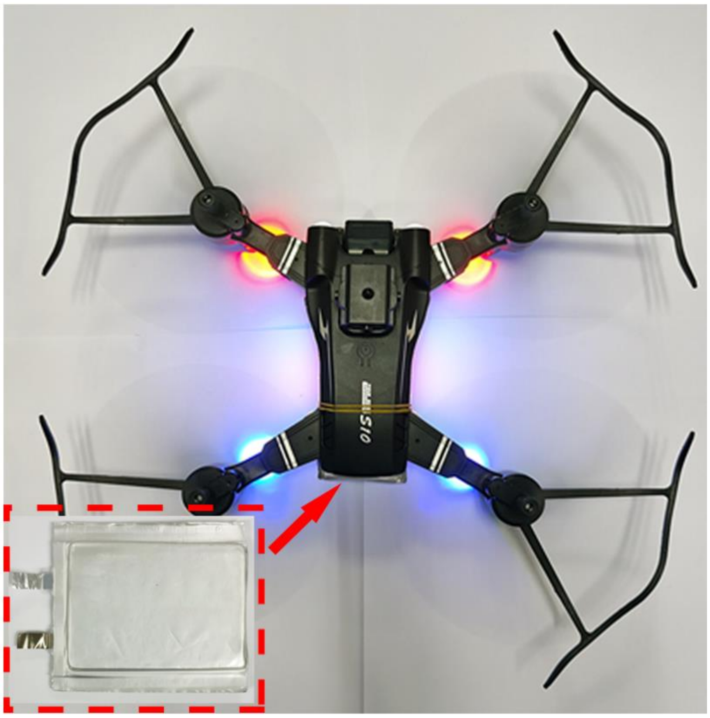

Fig. 31 The drone powered by the prepared pouch full cell

Supplementary References

[S1] M. Han, J. Li, J. Yu, Microspheres integrating Ti_2O_3 nanocrystals, carbon matrix, and vertical graphene enable fast ion transport for fast-charging lithium-ion batteries. J. Energy Stor. **43,** 103179 (2021). https://doi.org/10.1016/j.est.2021.103179

[S2] S.A. El-Khodary, G. Subburam, B.-B. Zou, J. Wang, J.-X. Qiu, X.-H. Liu, D.H. Ng, S. Wang, J.-B. Lian, Mesoporous silica anchored on reduced graphene oxide nanocomposite as anode for superior lithium-ion capacitor. Rare Met. **41,** 368-377 (2022). https://doi.org/10.1007/s12598-021-01788-z

[S3] J. Ma, J. Sung, Y. Lee, Y. Son, S. Chae, N. Kim, S.H. Choi, J. Cho, Strategic pore architecture for accommodating volume change from high Si content in lithium-ion battery anodes. Adv. Energy Mater. **10,** 1903400 (2020). https://doi.org/10.1002/aenm.201903400
